# Investigation of physical, structural, optical, and luminescence properties of nickel oxide doped alkali zinco-borate glasses

**DOI:** 10.1038/s41598-025-91852-w

**Published:** 2025-02-28

**Authors:** Vinay D., Devaraja C., R. S. Gedam, Eraiah B., G. V. Jagadeesha Gowda, Ashok Reddy G. V., Utpal Deka

**Affiliations:** 1 Department of Physics, Manipal Institute of Technology Bengaluru, Manipal Academy of Higher Education, Manipal, Karnataka 576104 India; 2 Department of Physics, Visvesvaraya National Institute of Technology, Nagpur, Maharashtra 440010 India; 3 Deparment of Physics, Bangalore University, Bengaluru, Karnataka 560056 India; 4 Department of Physics, Sambhram Institute of Technology, Bengaluru, Karnataka 560097 India; 5 Department of Physics, Nitte Meenakshi Institute of Technology, Bengaluru, Karnataka 560064 India; 6 Department of Physics, Sikkim Manipal Institute of Technology, Sikkim Manipal University, Majhitar, East Sikkim 737136 India

**Keywords:** Borate glasses, Deconvolution, Numerical aperture, Optical basicity, Photoluminescence, CIE chromaticity, Optics and photonics, Condensed-matter physics, Materials science, Materials for optics, Structural materials

## Abstract

Glasses having the stoichiometry ratio [(70-x) B_2_O_3_–10Na_2_O–20ZnO–xNiO] where 0.0 ≤ x < 0.3 mol% were synthesized using a melt quenching technique. The X-ray diffraction (XRD) technique confirmed the non-crystalline properties of the glasses. Surface morphology and elemental analysis were done by scanning electron microscope (SEM) and energy dispersive spectroscopy (EDS) spectra. The glass density ranged from 2.539 to 2.597 gcm^−3^ and the physical characteristics such as ion concentration, inter-ionic distance, average boron-boron distance, oxygen packing density, polaron radius, and field strength were calculated and interpreted. The deconvolution spectra of Fourier Transfer Infra-Red (FTIR) and Raman spectroscopy resulted in BO_3_ unit to BO_4_ unit conversion as the NiO concentration increased. The UV–Visible spectroscopy showed absorption peaks near 425 nm and 800 nm corresponding to ^3^A_2g_ (F) → ^3^T_1g_ (P)) and (^3^A_2g_ (F) → ^1^E_g_(^3^F)) transitions respectively. The direct bandgap decreased from 4.00 to 3.76 eV, but the indirect bandgap increased from 3.00 to 3.12 eV. The Urbach energy of glasses decreased from 0.56 to 0.35 with an increase in NiO concentration showing the compactness of the glass network. Furthermore, optical characteristics were determined, including the refractive index, dielectric constant, metallization criterion, electronic oxide ion polarizability, optical basicity, and numerical aperture. Photoluminescence spectra exhibit strong green and cyan emission due to d-d transitions of Ni^2+^. The CIE Chromaticity coordinates confirm that the observed green light emission from BZNNi glasses are suitable candidates for optoelectronic applications.

## Introduction

Borate glasses have gained importance in the field of technological investors and developers due to their electronic, thermal, optical, luminescent, and chemical resistance properties^1–5^. Because of its unique nature like non-toxicity, non-reactivity, and cost-effectiveness leads to industrial usage. B_2_O_3_ frequently coordinates with the three-oxygen called (BO_3_) units and four oxygen atoms called (BO_4_) units which are the building blocks of the glass network. Along with these two units B–O–B linkages inside the system can vary bridging oxygen (BO), non-bridging oxygen (NBO), and metal–oxygen bond interactions (M–O) with the addition of alkalis and metal oxides help to get distinct applications^4,6,7^. Conversely, zinc borate glasses gained significant attention because of their electrical, optical, and magnetic characteristics^3^. Zinc borate glasses have distinct qualities, such as their non-toxicity with strong chemical durability, which makes them suitable for the fabrication of optoelectronic devices, optical switching devices, lasing applications, and gas sensors^7–10^. Furthermore, zinc oxide can enhance the formation of various dopant sites within the borate glassy network, leading to enhanced optical and spectroscopic characteristics^11^. Additionally, the optical and electrical properties of sodium borate glasses are technologically appealing while making them suitable for incorporation into a wide range of electrical devices, including microwaves and electronic cells^12,13^.

Nickel oxide is one of the important transitional metals that are frequently used in a variety of potential applications, including solid oxide fuel cells, gas sensors, optical filters, and smart windows^14–16^. Glasses featuring nickel oxide are better in chemical stability, optical density, and durability. Because of its placement within the glass matrix and its ability to change the glass network, NiO can be utilized to color the host glass in small concentrations. In the glass matrix, nickel ions often occupy tetrahedral and octahedral sites.^17^. The quantitative features define the number of Ni^2+^ ions that occupy tetrahedral or octahedral sites^18^. There are few findings that have demonstrated the valuable impact of nickel addition on the growth of octahedral areas across the glassy network. Octahedral coordination is considerably preferred to tetrahedral coordination in glasses containing Ni^2+^ ions, due to its large crystal field stabilization energy^19^. Many unique and robust absorption bands can be found in the visible and near-infrared spectrums when nickel ions are octahedrally coordinated. Consequently, in the field of laser technology, these glasses can be an essential tool for amplification^20^.

Abdelhamied et al.^21^ investigated the effect of NiO doping and nitrogen (N) ion beam irradiation on structural compactness was examined, with density ranging from 2.47 to 3.9 g/cm^3^ and bandgap decreasing from 2.76 to 2.61 eV. It also increased the dielectric characteristics, resulting in photodetector and optoelectronic device applications. Seema Thakur et al.^22^ have studied the nickel-doped bismuth borate glasses. The density ranged from 6.812 to 8.301 g/cm^3^. The ionic nature of diseases in glass networks, and the optical properties of the glasses, assist this, resulting in feasible applications. Abul-Magd et al.^13^ synthesized NiO-doped borate glasses. The density ranged from 2.587 to 2.746 g/cm^3^, and the structural and optical properties demonstrated the semiconductor behavior of the produced glass samples. Nada Alfryyan et al.^23^ examine the influence of NiO-doped boro-phosphate glasses, based on their physical, structural, and gamma-ray attenuation properties. Both the density and the optical bandgap decreased from 2.52 to 2.66 g/cm^3^ and 3.88 eV to 3.49 eV, respectively. The prepared glass is capable of replacing commercial concrete for shielding. Based on the best available literature, an extensive amount of research has been done on how NiO affects glass materials, but different chemical compositions with low nickel concentrations for solid-state and optoelectronic light applications have not yet been explored in the study of glass properties.

In the present work, we have investigated a novel composition of the influence of low NiO concentration on the physical, structural, morphological, optical, and photoluminescence (PL) properties of alkali zinc borate glasses using XRD, FTIR, Raman, SEM, EDS, UV–VIS, and PL spectroscopy respectively. The calculated values of optical properties with PL emission spectra and CIE Chromaticity analysis connect the results to the practical applications in optoelectronic devices.

## Experimental details

### Sample preparation

**Fig. 1:**
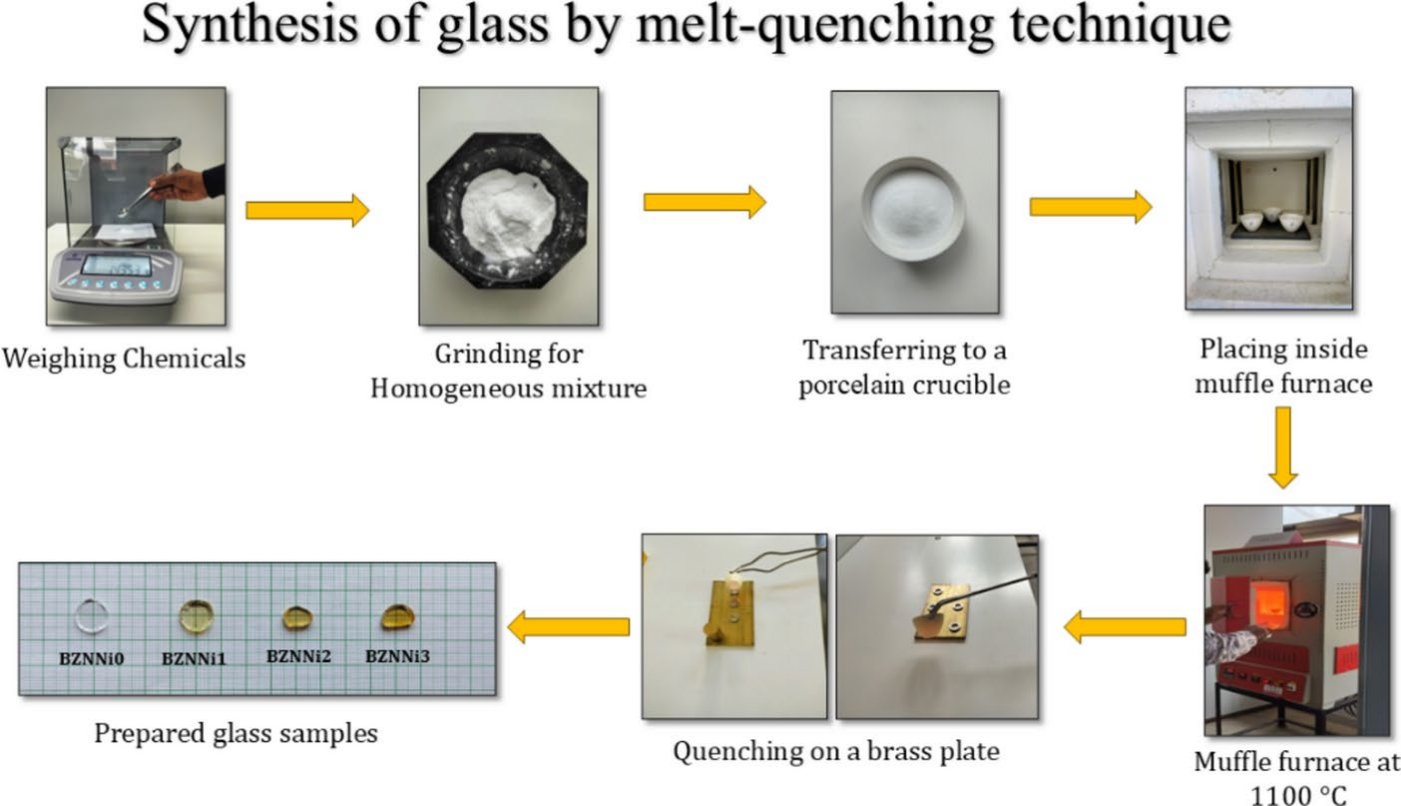
Schematic representation of the BZNNi series glass.

The present glassy system with a quaternary [(70-x) B_2_O_3_–10 Na_2_O–20 ZnO–x NiO], where 0.0 ≤ x < 0.3 mol% with 0.1 mol% increments was prepared via the melt quenching method and coded as shown in Table 1. The raw ingredients comprised 99.99% pure boric acid (H_3_BO_3_), zinc oxide (ZnO), sodium carbonate (Na_2_CO_3_), and nickel sulphate (NiSO_4_) from Sigma Aldrich. Chemicals were weighed precisely according to the stoichiometry ratio and completely grinded using an agate mortar and pestle to get homogeneity in the mixture. Then the prepared chemicals were transferred to the porcelain crucible, placed inside the muffle furnace, and heated to 1100 °C. The obtained super-cooled liquid is poured onto a brass plate with stainless steel rings on it and then pressed immediately with another brass mold to get the flat circular pellet samples as shown in Fig. 1. These molds were annealed at 350 °C to reduce the thermal stress of the glass while cooling. To avoid moisture ingestion from the air, the samples were stored in a desiccator. The thickness of the glass is found to be 0.247 cm, 0.273 cm, 0.271 cm, and 0.294 cm and the diameter of the glass is 1.288 cm, 1.25 cm, 0.98 cm, and 1.1 cm for glass coding BZNNi0, BZNNi1, BZNNi2, and BZNNi3 respectively.

**Table 1:** Composition of the glass samples.

Sample composition				
Sample code	B_2_O_3_ mol%	ZnO mol%	Na_2_O mol%	NiO mol%
BZNNi0	70	20	10	0.0
BZNNi1	69.9	20	10	0.1
BZNNi2	69.8	20	10	0.2
BZNNi3	69.7	20	10	0.3

### Sample characterization

Archimedes’ buoyancy effect principle was used to determine the density of the prepared glass samples. Toluene was used as a suspension medium at room temperature The powder sample’s non-crystallite nature was analyzed by X-ray diffraction (XRD) utilizing an XRD RIGAKU with a 6000-type diffractometer, a Cu radiation source (λ = 1.541 Å), and 1200 watts of power. The ZEISS EVO 10 Scanning Electron Microscope (SEM) equipped with optional backscatter detection and an Element Energy Dispersive Spectroscopy (EDS) system is used to analyze the morphological and elemental composition of the glass samples. A thin layer of gold sputtering was coated on the sample’s surface to reduce the charge effects caused by the electron beam. The infrared absorption spectra of crushed and finely powdered samples were recorded at room temperature using an ATR-FTIR-type spectrometer ranging from 500 to 1600 cm^-1^. The Raman spectra are obtained by Horiba Jobin Yvon-Xplora plus V2.1 multiline confocal Raman microscope 200–1600 cm^−1^. The samples were polished to a smooth surface using emery paper with a grit size of 320 to 1500 microns to record the optical spectra. UV–visible absorption spectra (200–1100 nm) were obtained at room temperature using a Shimadzu UV-1800 spectrometer. The powdered glass samples were used in the PL-100-CC with a fluorescence CCD array-based spectrometer with a 240–850 nm excitation and 200–900 nm emission range to obtain the PL spectra.

## Result and discussion

### X-ray diffraction (XRD) analysis

**Fig. 2:**
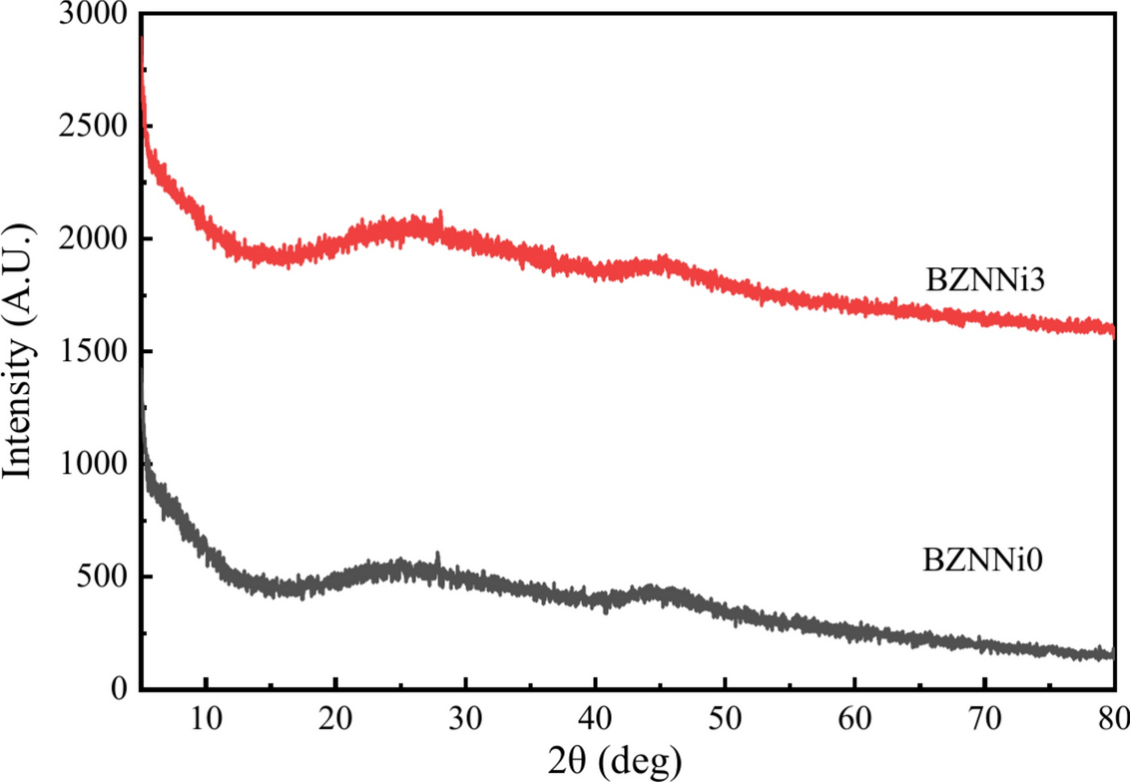
Typical XRD pattern of the BZNNi0 and BZNNi3 glasses.

Figure 2 shows the X-ray diffracted patterns of BZNNi0 and BZNNi3 obtained at room temperature at a 2° per minute scanning rate between 5° and 80°. The presence of broad humps represents the scattered structure of the short-range orders in the network. Broad hump in the 15°–35° and 40°–50° regions suggests the sample’s long-range disordered characteristics due to the presence of metallic cations, oxygen with other network forming and modifying units in the glass networks^24,25^. This clearly illustrates a non-crystalline structure in the synthesized glass samples.

### Scanning Electron Microscope (SEM) and Energy Dispersive Spectroscopy (EDS)

**Fig. 3:**
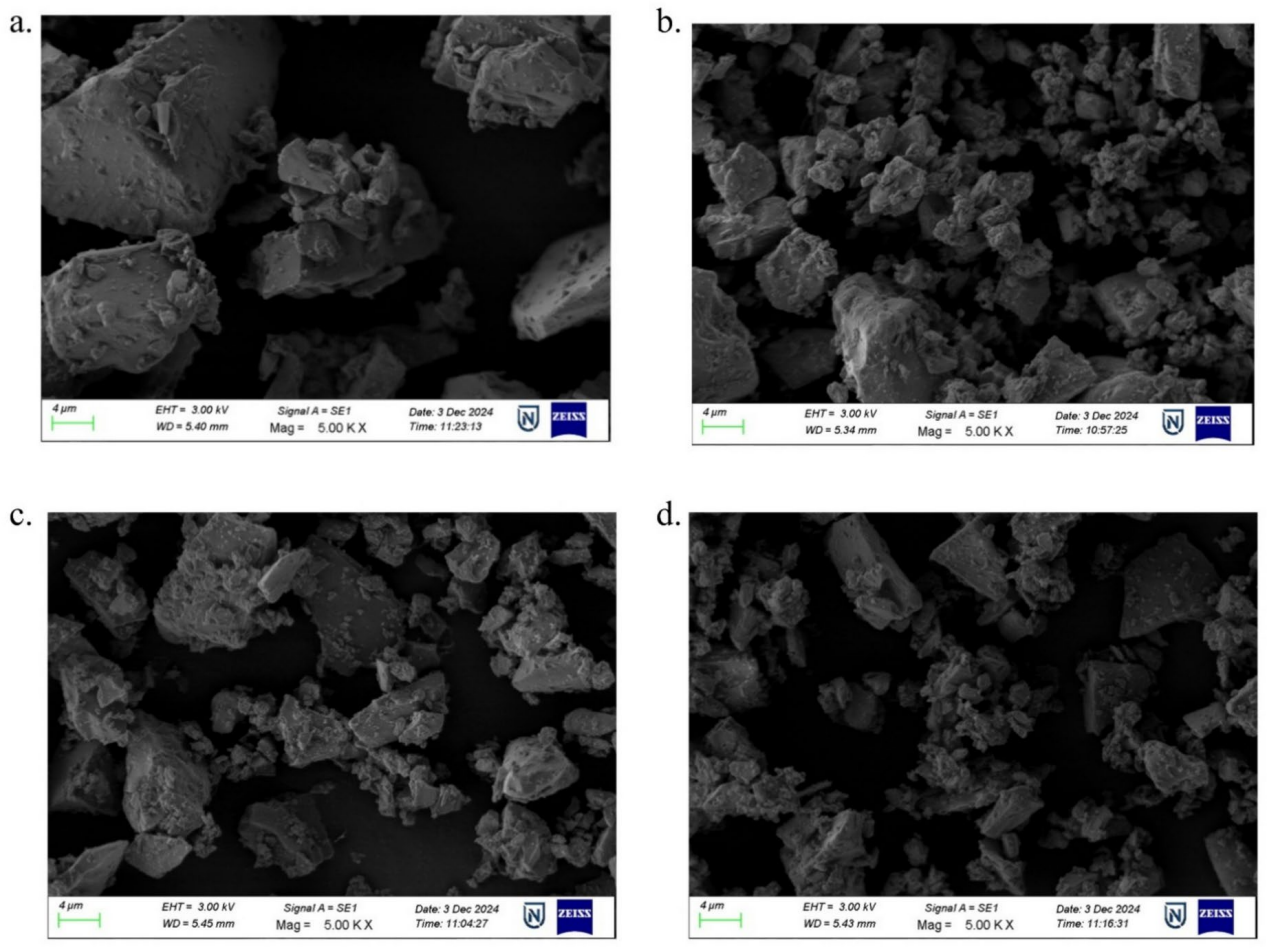
The SEM photographs of (**a**) BZNNi0. (**b**) BZNNi1. (**c**) BZNNi2. (**d**) BZNNi3 glasses.

**Fig. 4:**
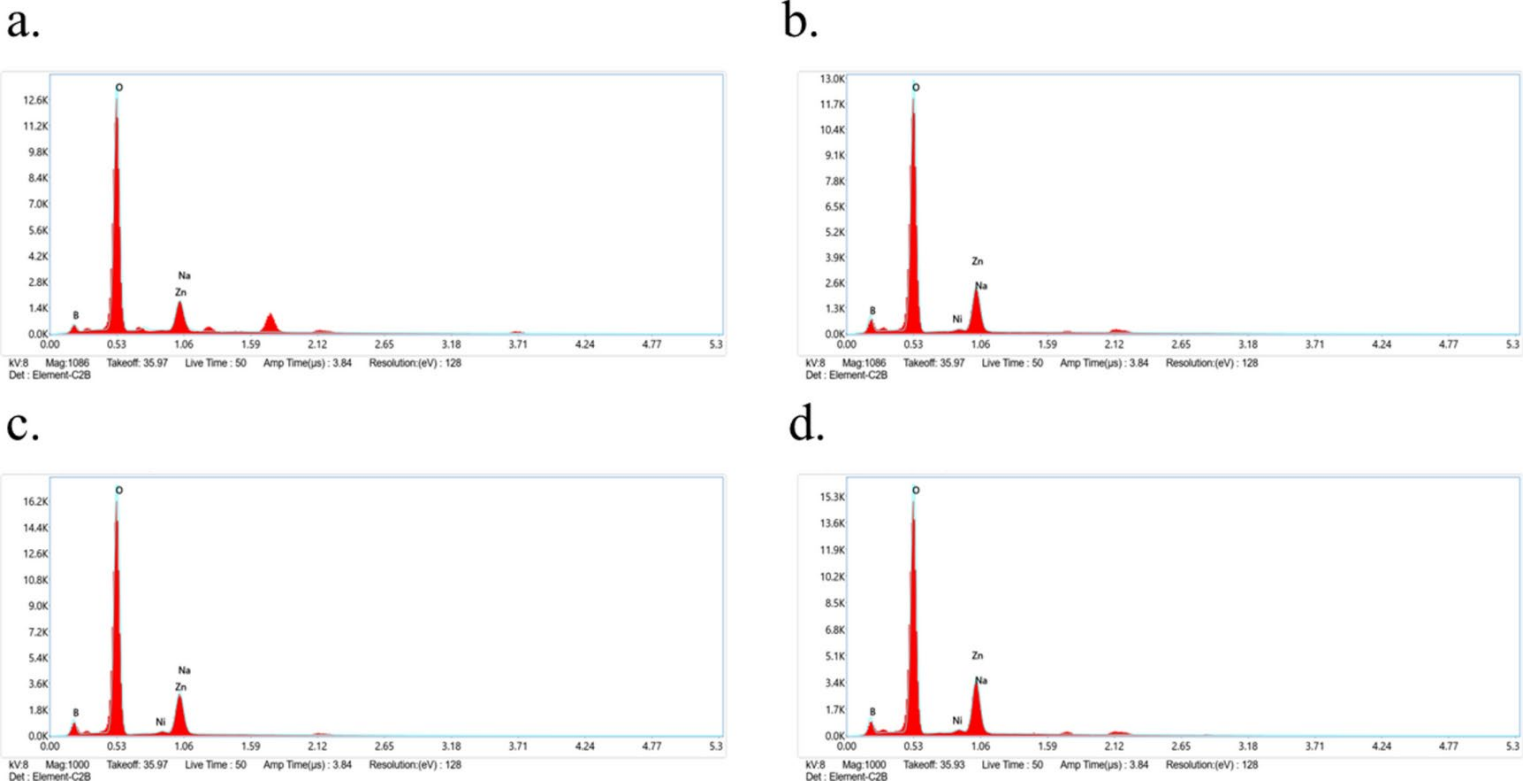
The EDS spectra of (**a**) BZNNi0 (**b**) BZNNi1. (**c**) BZNNi2. (**d**) BZNNi3 glasses.

SEM images and EDS spectra are essential for characterizing the material to gain surface-level morphological analysis and the elemental composition of the samples. They are beneficial in identifying the crystal structure in the prepared samples. Figure 3 display the SEM images of the prepared BZNNi glasses, where these particles are placed under a high vacuum with a 5.00 KX magnification level. Neither of the BZNNi glass samples displayed crystal structure due to the absence of long-range periodic distribution of the particles in the samples. BZNNi glass powder is visible in the pictures as particles with distinct sizes of a few hundred nanometers. When these nanometric dust particles are magnified by 5.00 KX, it is possible to identify amorphous chemical configurations on the particles that do not have an ordered crystal structure. Figure 4. illustrates the EDS spectra of BZNNi samples. When nickel oxide was added to the structure, the EDS spectrum for each sample showed an increase in the peak associated with Ni. Each sample exhibits B, Zn, Na, and O peaks with high intensity confirming its presence. Nevertheless, the EDS spectra data do not show a regular distribution based on the area where the measurement was taken. This demonstrates that the amorphous structure and not truly homogeneous. These non-homogeneous have impactful effects on the physical, structural and optical properties. These properties will vary due to their unlikely distribution of the dopants in the base glass leading to a change in structure. Furthermore, a change in structural properties leads to a change in physical and optical properties which is discussed below section. The absence of distinct elements indicated that the samples were pure since no external substances from the porcelain crucible were incorporated into the glass samples^26–28^.

### Physical properties

**Fig. 5:**
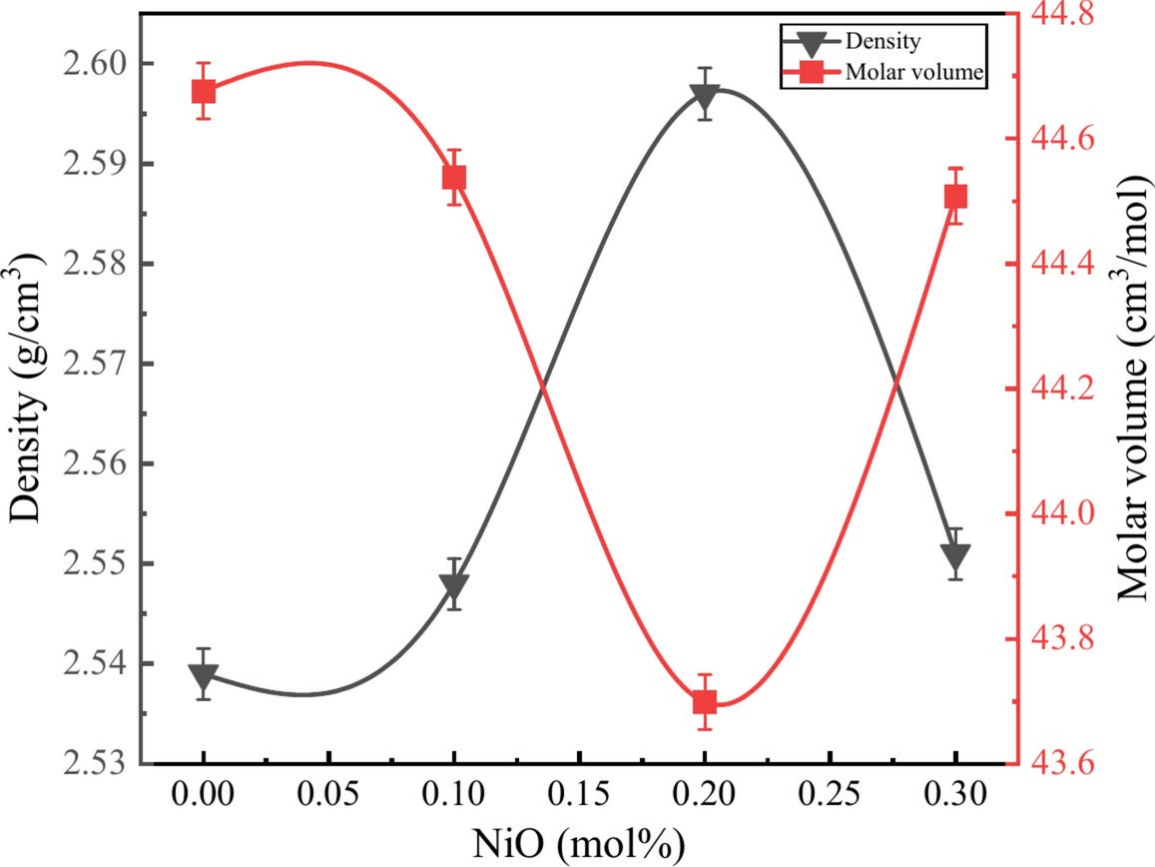
Relationship between $\rho$ and ${V}_{m}$ of the BZNNi glass series.

Density is essential for understanding structural changes in glass systems. The geometrical arrangement, compactness, glass interstitial space dimensions, and coordination number are a few basic variables that influence the density of the glass system. Equation 1 is used to determine the density of the synthesized glass samples based on the Archimedes Principle^22,29^.

1 $$\rho =\frac{{w}_{a}}{{w}_{a}-{w}_{l}}{\rho }_{l}$$

where $${\rho }_{l}$$ is the density of the liquid used (toluene = 0.867 gcm^-3^), $${w}_{a}$$ is the glass sample weight in the air, and $${w}_{l}$$ is the glass sample weight in liquid. Following that, molar volume ($${V}_{m})$$ of glass samples were calculated using Eq. 2^30^.

2 $${V}_{m}=\frac{{M}_{w}}{\rho }$$

where ${M}_{w}$ and $\rho$ are the molecular weight and density of the glass sample, respectively. The change in the density and molar volume of the BZNNi series glass as the NiO mol% increases as shown in Fig. 5 and shown in Table 2. The value of the density initially increased from 2.539 to 2.597 g/cm^3^ and the molar volume decreased from 44.676 to 43.699 cm^3^/mol from 0 to 0.2 mol%. This nature indicates that Ni ions filled the crevice of the borate network by forming B-O-Ni linkages in glass structures. The sudden drop in density to 2.551 g/cm^3^ and increase in the molar volume to 44.508 cm^3^/mol at 0.3 mol% refers to the Ni ions no longer filling the crevice of the glass network indicating the formation of Ni–O–Ni linkages.^11,31,32^.

**Table 2:** Density ($\rho )$, molar volume (${V}_{m}$), ion concentration (${N}_{i}$), inter ionic distance (${r}_{i}$), average boron–boron distance ($<B-B>$), oxygen packing density ($OPD$), polaron radius (${r}_{p}$), field strength ($F$) (All parameters were determined with an error of ± 0.001).

Sample code	$\uprho$(g/cm^3^)	${\text{V}}_{\text{m}}$(cm^3^/mol)	${\text{N}}_{\text{i}}$ (ions/cm^-3^ × 10^19^)	${\text{r}}_{\text{i}}$(nm)	< *B-B* > (Å)	$OPD$, (g.atom/l)	${r}_{p}$(nm)	$F$(cm^-2^ × 10^14^)
BZNNi0	2.539	44.676	0	0	4.982	53.720	0	0
BZNNi1	2.548	44.538	2.704	3.332	4.971	53.842	1.340	1.109
BZNNi2	2.597	43.699	5.512	2.628	4.934	54.829	1.060	1.783
BZNNi3	2.551	44.508	8.118	2.309	4.959	53.788	0.931	2.309

**Fig. 6:**
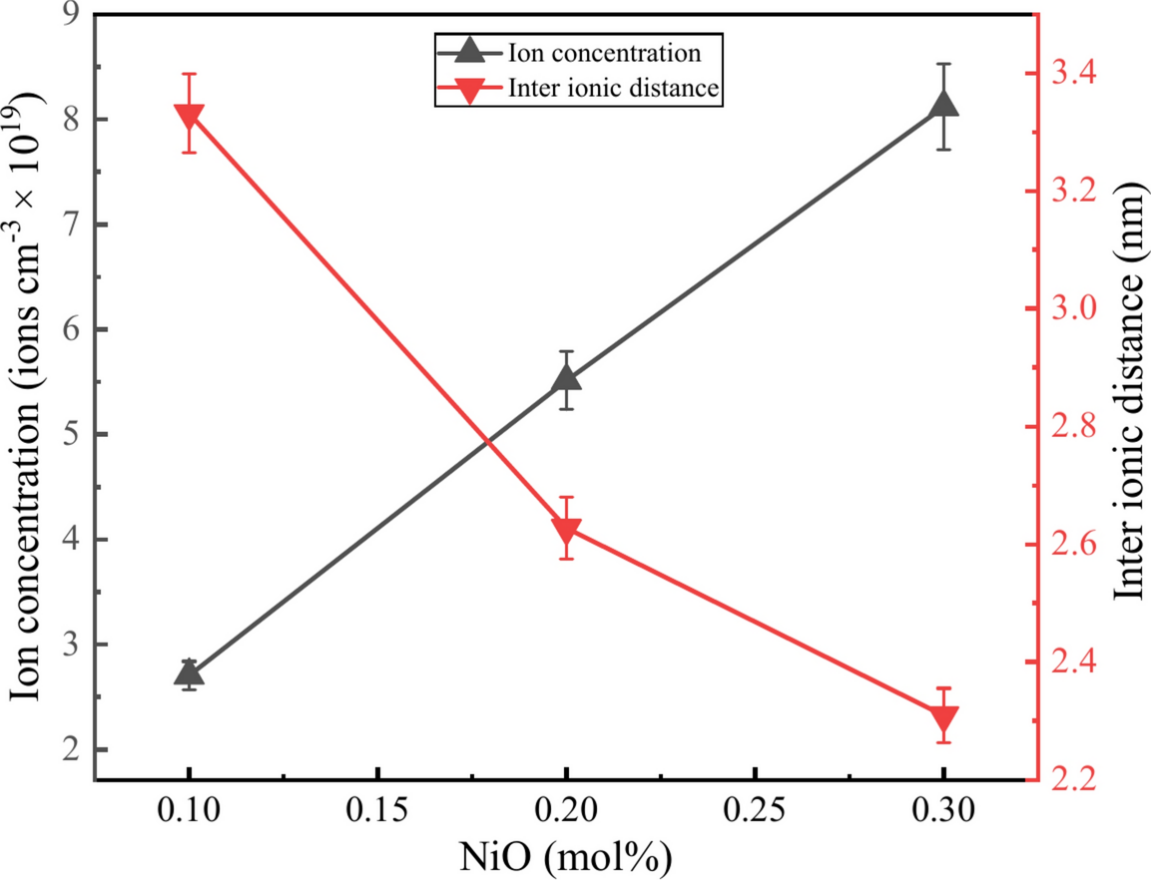
Relationship between ${N}_{i}$ and ${\text{r}}_{\text{i}}$ of the BZNNi glass series.

The ion concentration (${N}_{i}$)^33^, inter ionic distance (${r}_{i}$)^33^, average boron-boron distance $(<B-B>$)^34^, oxygen packing density ($OPD$)^35^, polaron radius (${r}_{p}$), field strength ($F$)^34^ of the prepared glasses is calculated using the following equations.

3 $${N}_{i}=\frac{{N}_{A} \times mol\text{\% }of\, cation\, \times valance\, of\, cation}{{V}_{m}}$$

4 $${r}_{i}={(1/{N}_{i})}^{1/3}$$

5 $$\left\langle {B - B} \right\rangle = \left( {\frac{{V_{m} }}{{2N_{A} \left( {1 - X_{B} } \right)}}} \right)^{\frac{1}{3}}$$

6 $$OPD=\frac{1000\times O}{{V}_{m}}$$

7 $${r}_{p}=\frac{1}{2}{(\frac{\pi }{{6N}_{i}})}^\frac{1}{3}$$

8 $$F=\frac{Z}{{({{\varvec{r}}}_{{\varvec{p}}})}^{2}}$$

where, ${N}_{A}$ is the Avagadro’s number, ${X}_{B}$ represents mol% of the B_2_O_3_, $O$ is the number of oxygens in the glassy network, and $Z$ represents the valence of the cation doped to the glass. As Ni^2+^ concentration increased the ${N}_{i}$ tends to increase but ${r}_{i}$ decreases as the Ni^2+^ replaces the boron atom in the glass system. The trend is due to an increase in NiO concentration, the nickel ions in the network coming closer to each other and reducing the distance between nickel ions and increasing its ionic concentration inside the glass network, as shown in Fig. 6 due to the addition of the excess NiO in the borate glass network^36^. Contrary the < B-B > decreased to 4.934 Å from 4.982 Å and OPD increased to 54.829 g.atom/l from 53.720 g.atom/l till 0.2 mol% later increased in < B-B > up to 4.959 Å and decrease in OPD up to 53.788 g.atom/l shows the same trend as the $\rho$ and ${V}_{m}$. This is because the B–O–Ni and Ni–O–Ni linkages formed in the glass network. Furthermore, the increase in the polaron radius due to excess of nickel ions which makes the distance closure, and the field strength can be seen due to an increase in the concentration of Ni^2+^ in the glass network^11^. The calculated value is shown in Table 2.

### Fourier transform infrared (FTIR) spectroscopy

**Fig. 7:**
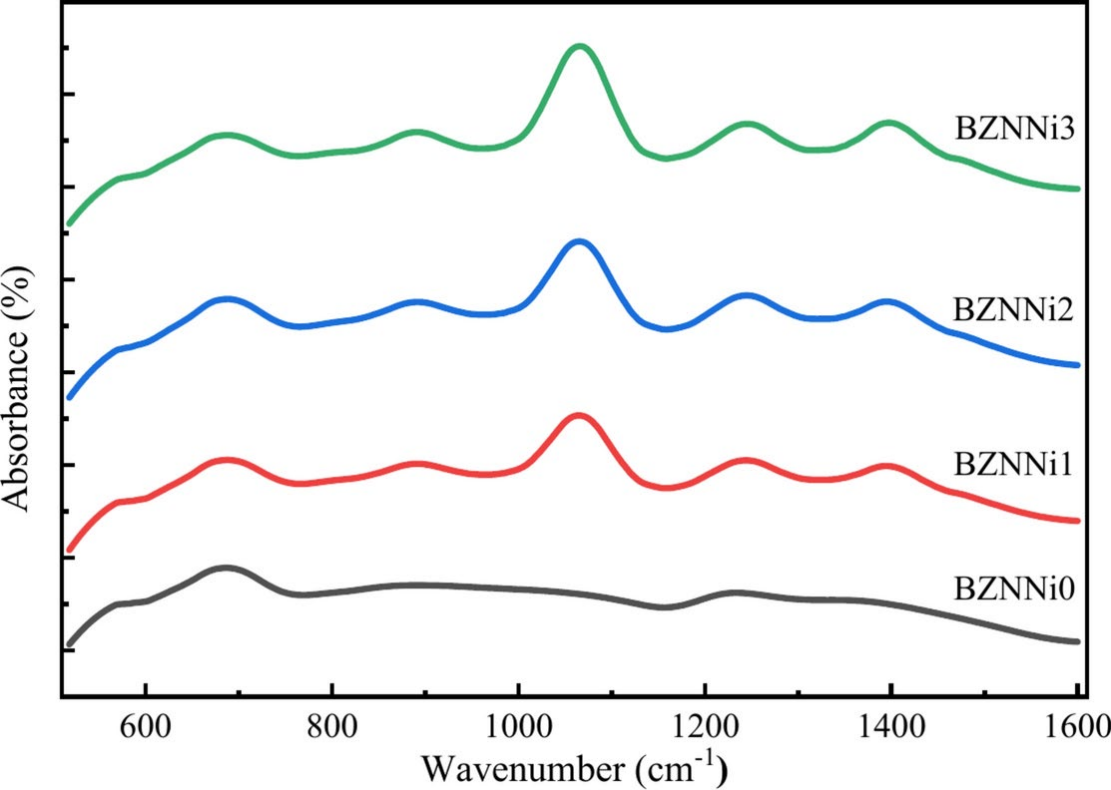
FTIR spectra of the BZNNi glass series.

Vibrational spectroscopy is an effective and non-destructive technique that can interpret the structural composition of the functional group present in the prepared glass network. IR spectroscopy provides information about the presence of alkali, and transition metal cations including the interactions in glass matrices like intermediate range ordered boroxol rings (B_3_O_6_), and basic structural units like tetrahedral (BO_4_) and triangular (BO_3_) short-range structures. The FTIR spectra of synthesized glass matrix coded BZNNi0, BZNNi1, BZNNi2, and BZNNi3 were obtained at room temperature in the 500–1600 cm^-1^ range as shown in Fig. 7. The FTIR spectra of boronated glass can be divided into three major IR zones. The first zone explains the symmetric and asymmetric vibrations of the BO_3_ units from the range of 1200–1600 cm^-1^. The tetrahedral BO_4_ units exhibit symmetric and asymmetric vibrations in the second zone, ranging between 800 and 1200 cm^-1^. Finally, the third zone, which ranges from 600 to 800 cm^-1^, explains the B-O-B of BO_3_ and BO_4_ units bending vibration.

**Fig. 8:**
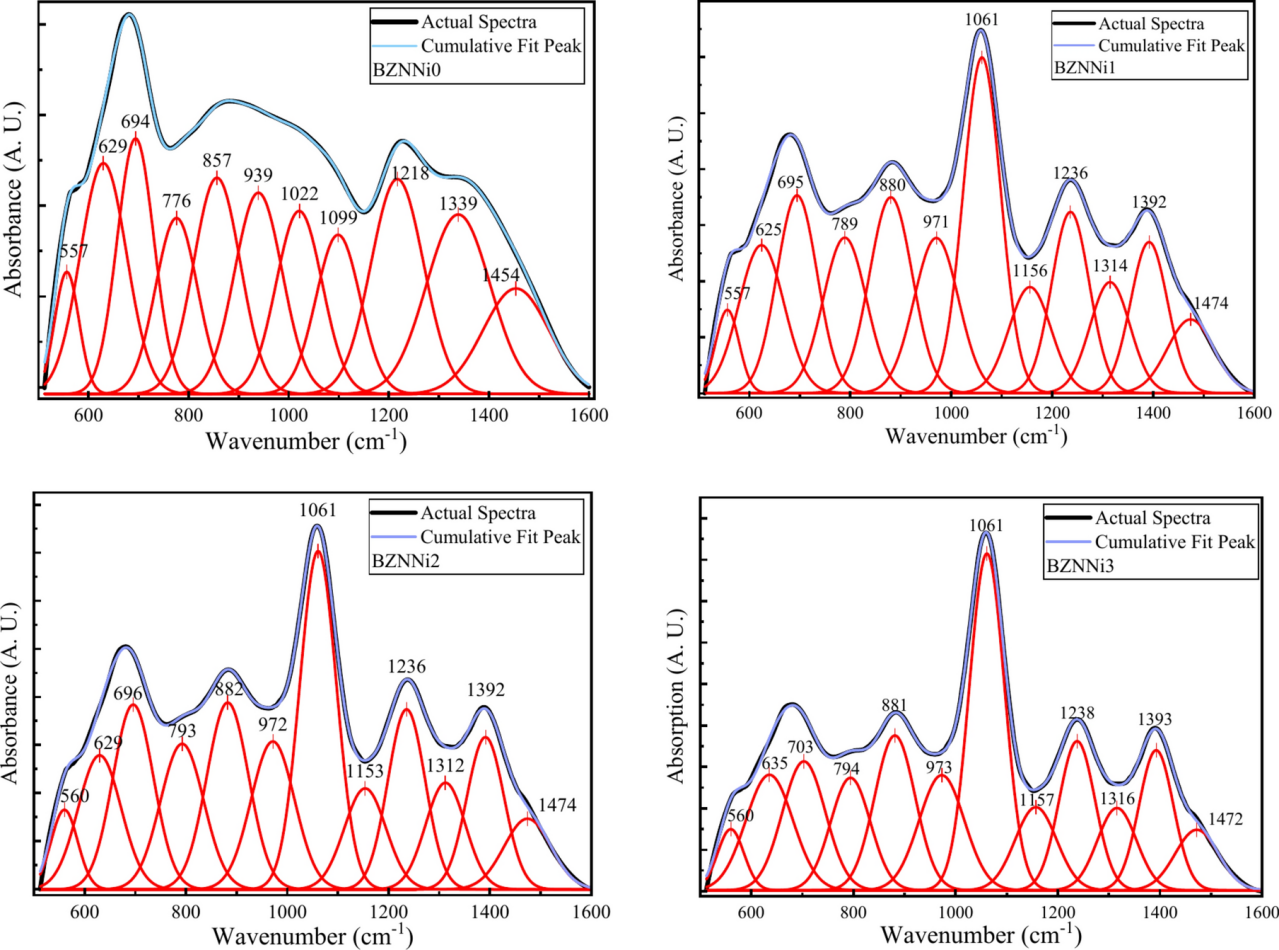
Deconvoluted FTIR spectra of the BZNNi glass series.

To analyze the presence of the diverse segments of the borate structures, deconvolution of the FTIR spectra is performed using the Origin 2024 software in the built-in function ‘Multiple Peak Fit’ with an R^2^ value of 0.99. Deconvolution separates the superposition of the distinctive band structures. Broad and asymmetric absorption bands of the FTIR spectra on the investigation in the range given above are explained in reference to Fig. 8 and the band assignment is displayed in Table 3. The peak in the range 557–560 cm^-1^ represents the vibration of Na^+^ cations^37,38^. The deconvoluted broad peak ranging from 600 to 770 cm^-1^ contains 629–635 cm^-1^ and 694–703 cm^-1^ due to B-O-B bending vibration present in BO_3_ units and Ni–O–Ni linkages in the network^39^. The small hump near 776–794 cm^-1^ is associated with bridging oxygen bonds between BO_3_ triangular units^40^. The peaks from BZNNi0 at 857 cm^-1^ represent di-borate linkage^38^ in borate networks in B-O-B form, but in BZNNi1, BZNNi2, BZNNi3 880 cm^-1^, 882 cm^-1^, 881 cm^-1^ represents triborate, tetraborate and pentaborate groups of B–O–B bending vibrations due to incorporation of the nickel oxide into the glass^38,41^. ~ 970 cm^-1^ and ~ 1061 cm^-1^ in BZNNi1, BZNNi2, BZNNi3 and ~ 939 cm^-1^ in BZNNi0 is due to vibration of the B–O bonds in the BO_4_ group due to the presence of di-borate in the system, the intensity of the peak at 1061 cm^-1^ increases represents at small quantity if the nickel in the glass system acts as the glass network former instead of modifier and 1022 cm^-1^ and 1099 cm^-1^ in BZNNi0 explains about B-O symmetric stretching of BO_4_ units due to presence of triborate, tetraborate and pentaborate groups in the system^42–44^. The peak ~ 1150 cm^-1^ and ~ 1230 cm^-1^ in BZNNi1, BZNNi2, BZNNi3, and 1218 cm^-1^ represent stretching vibrations in B-O bonds of BO_3_ units of pyro- and orthoborate groups in the glass system^38,41^. The peak ~ 1340 cm^-1^ in BZNNi0 and the peaks near ~ 1315 cm^-1^ and ~ 1390 cm^-1^ represent the pyro- and ortho-borate groups containing BO^3-^ (NBOs) and various borate rings vibrations of B-O in BO_3_ units splitting of the peaks due to presence of the NiO in the system^40,45^. The peaks 1454 cm^-1^ and 1470 cm^-1^ refer to asymmetric stretching modes of triangles formed from borate. NiO in the system is the cause of the shift in peaks in BO_3_ units^46^. The N4 parameter is calculated by using N4 = (area under A of BO_4_ units)/((area under A of BO_4_ units) + (area under A of BO_3_ units)). The calculated values of N4 are found to be 0.54, 0.60, 0.62, and 0.63 for BZNNi0, BZNNi1, BZNNi2, and BZNNi3 glass samples respectively^29^. The increase in the N4 value leads to the formation of the BO_4_ units from BO_3_ units. This conversion leads to compactness of the glass where at less concentration NiO in the glass acts as glass former rather than a modifier.

**Table 3:** FTIR band assignments of BZNNi glass series.

BZNNi0	BZNNi1	BZNNi2	BZNNi3	Band Assignments	Ref
557	557	560	560	Vibration of Na^+^ cations	^37,38^
629	625	629	635	Bending vibration of B-O-B linkages present in BO_3_ units and Ni–O–Ni linkages	^22,39^
694	695	696	703		
776	789	793	794	Bridging oxygen bonds between BO_3_ and [BO_4_] units	^13,40^
857				Di-borate linkage in B–O–B networks	^38^
	880	882	881	Tri-borate, tetra-borate and penta- borate groups of B–O–B bending vibrations	^38,41^
939	971	972	973	Di-borate vibrations in BO_4_ units by B–O bonds	^42–44^
1022	1061	1061	1061	B–O symmetric stretching of BO_4_ units due to the presence of tri-borate, tetra-borate, and penta-borate groups	
1099					
	1156	1153	1157	B–O stretching vibrations in BO_3_ units of pyroborate and orthoborate groups	^38,41^
1218	1236	1236	1238		
1339	1314	1312	1316	Pyro-borate and ortho-borate groups containing BO^3-^ (NBOs) and various borate ring vibrations of B-O in BO_3_ units splitting of the peaks	^40,45^
	1392	1392	1393		
1454	1474	1474	1472	Asymmetric stretching modes of triangles formed from BO_3_ units	^46^

### Raman spectroscopy

**Fig. 9:**
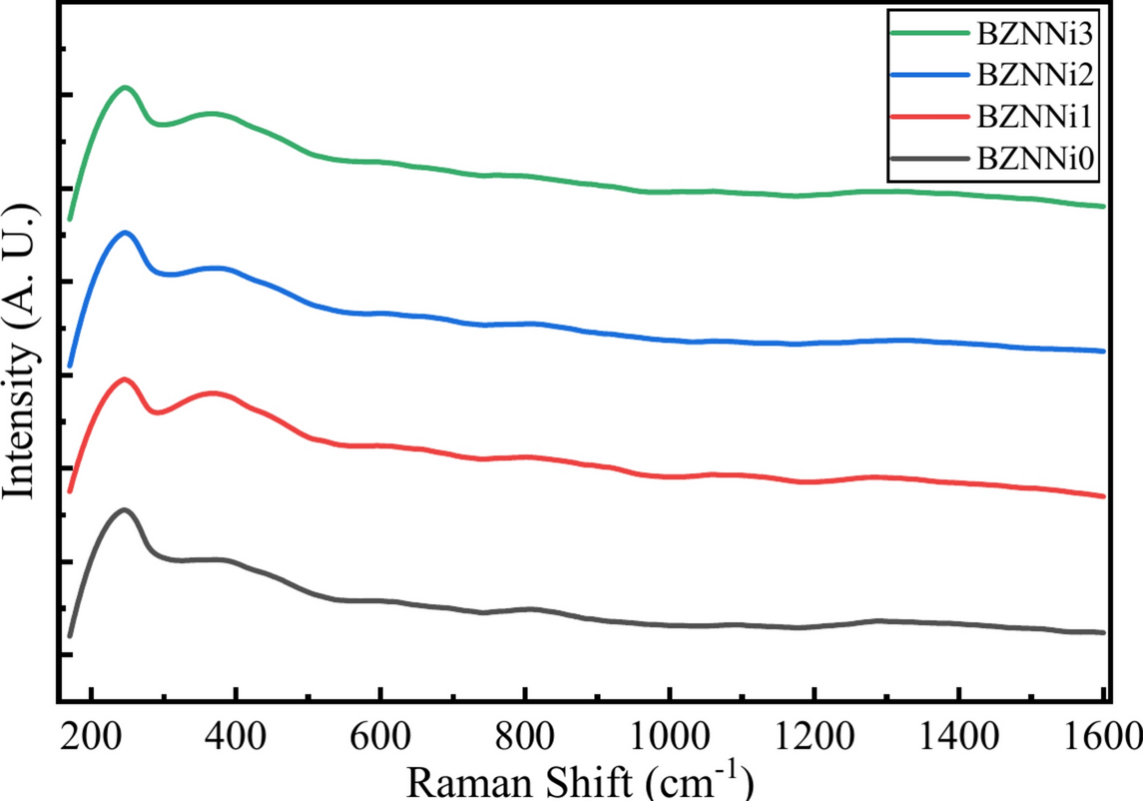
Raman spectra of the BZNNi glass series.

**Fig. 10:**
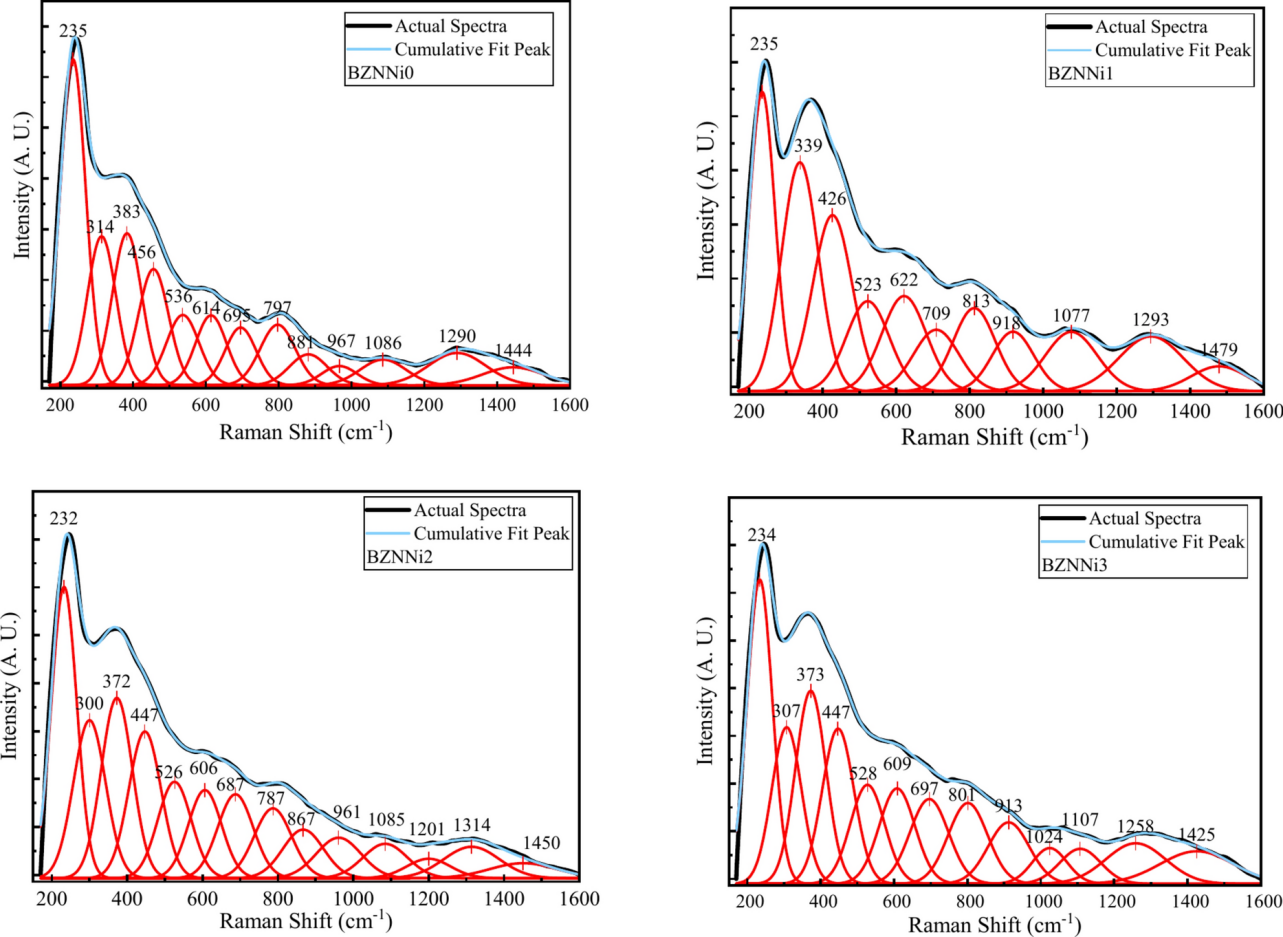
Deconvoluted Raman spectra of the BZNNi glass series.

Figure 9 displays the Raman spectra of the synthesized glass samples. There are hidden peaks within the broad spectrum in the obtained Raman spectra. The deconvoluted peaks, which are illustrated in Fig. 10, and band assignments are shown in Table 4. The Raman spectra clearly show three regions: (I) 200–600 cm^-1^, (II) 600–1200 cm^-1^, and (III) 1200–1600 cm^-1^. The peaks represent the ZnO_4_ unit’s bending vibrations and the Na^+^ and Ni^2+^ cationic vibrations in the range 235–339 cm^-1^ found in the prepared glass samples^25,47,48^. Raman peaks at 373–426 cm^-1^ correspond to isolated diborate groups^38^. The band at 480–700 cm^-1^ corresponds to the overlapping of B-O-B bending vibrations and the BO in the Ni–O–Ni linkage^25,36^. The spectral range 480–700 cm^-1^ indicates the presence of ring-type metaborate units^48^. A band seen in the 787–813 cm^-1^ range is expected to be caused due to the vibrations of rings that contain BO_3_ (triangular) and BO_4_ (tetrahedral) units^37^. B–O stretching vibrations in the ortho-borate group are indicated by BO_4_ units in the band ranging from 881 to 967 cm^-1^. The presence of tetra, penta, and di-borate groups is subjected to the Raman bands observed in the spectral range of 1024–1202 cm^-1^ due to the presence of Ni^2+^ in the glass system influencing the variation of intensity of the spectra^37,49^. Peaks around 1258–1322 cm^-1^ are associated with BO_3_ units in meta, orthoborate, and pyroborate with B–O stretching vibrations^25^. The peak in the range 1425–1465 cm^-1^ is caused by B–O^-^ vibrations from BO_3_ units, which connect to a large borate network^47^.

**Table 4:** Raman band assignments of BZNNi glass series.

BZNNi0	BZNNi1	BZNNi2	BZNNi3	Band Assignments	Ref
235	235	236	234	The bending vibrations of ZnO_4_ units and vibrations of Na^+^ and Ni^2+^ cations	^25,47,48^
314	339	332	307		
383	426	420	373	Isolated diborate groups	^38^
456			447	Overlapping of B–O–B bending vibrations and the Ni–O–Ni linkage	^25,36^
536	515	507	528		
614	604	611	609		
695	690	700	697		
787	800	813	801	Vibrations of rings that contain BO_3_ and BO_4_ units	^37^
881	910	934	913	B–O stretching vibrations in the ortho-borate group by BO_4_ units	^37,49^
967					
1086	1029	1078	1024	The presence of tetra, penta, and di-borate groups	
	1112	1202	1107		
1290	1283	1322	1258	Stretching vibrations of BO_3_ units in meta, orthoborate, and pyroborate	^25^
1444	1465	1438	1425	B–O^-^ vibrations from BO_3_ units	^47^

### Optical properties

**Fig. 11:**
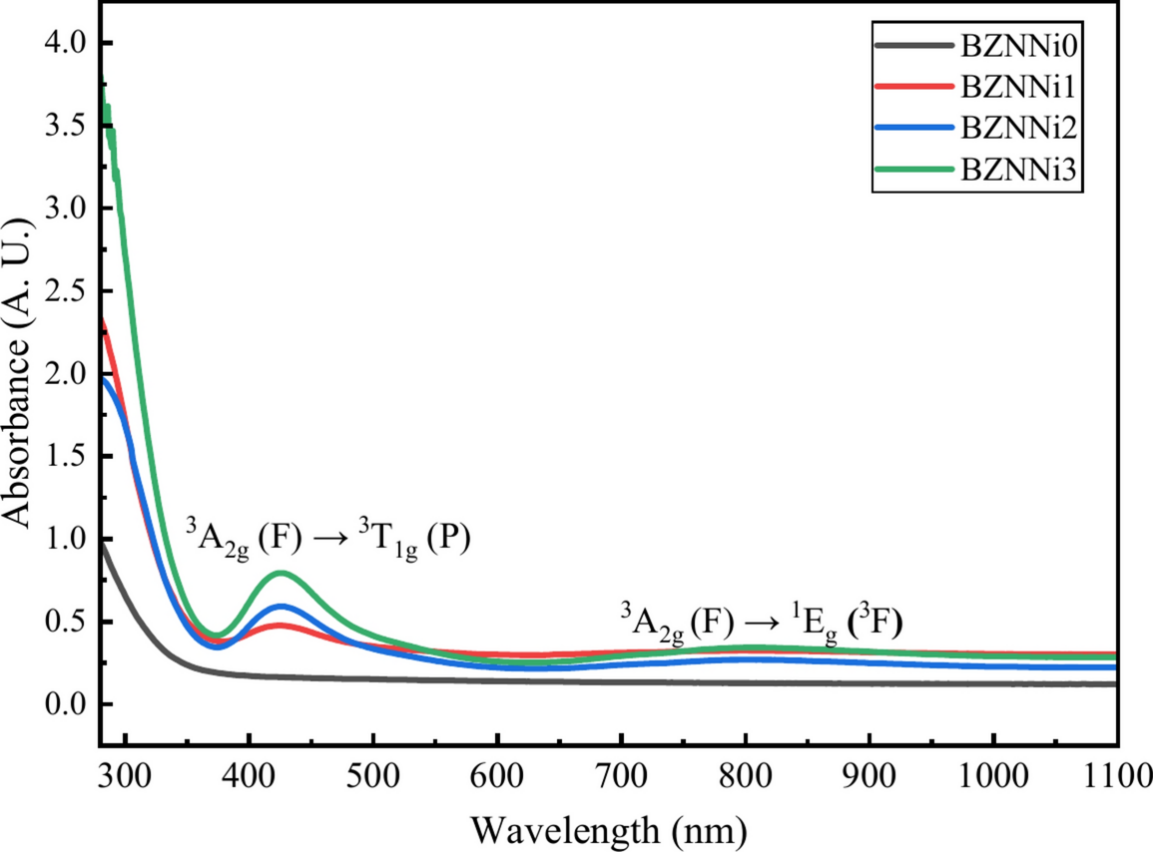
Absorption of UV–visible spectra of the BZNNi glass series.

Optical absorption spectra are a valuable approach for analyzing electronic structures in glass medium. Figure 11 displays the absorption of optical spectra of prepared glasses. Redshift, or the change in the cutoff wavelength to higher wavelengths is noticed when the nickel content increases. When NiO was added to the glass, the absorption increased, resulting in an absorption band at 425 nm and a broad absorption band at 800 nm. The transition of Ni^2+^ ions in octahedral coordination is ascribed to the spin-allowed transition ^3^A_2g_ (F) → ^3^T_1g_ (P) (425 nm) and the spin-forbidden transition ^3^A_2g_ (F) → ^1^E_g_(^3^F) (800 nm) of Ni^2+^ in octahedral coordinates^40,50^. Furthermore, as the NiO concentration increases, absorbance increases. This implies a band-shrinking effect, in which the number of electrical transitions to higher energy levels rises. With more nickel present, more photons interact with the material, resulting reduction in the optical bandgap^51^.

### Optical bandgap energy (${{\varvec{E}}}_{{\varvec{o}}{\varvec{p}}{\varvec{t}}}$)

Analysis of the electronic transitions in materials is done using the traditional method. Equation 9 is used to determine the coefficient of optical absorption (α)^52^. The optical bandgap values are displayed in Table 5.

9 $$\alpha =2.303 \times \frac{A}{T}$$

where $T$ is the thickness of the glass sample and $A$ is the absorbance.

**Table 5:** Optical properties of the BZNNi glass series (All parameters were determined with an error of ± 0.0001).

Sample code	BZNNi0	BZNNi1	BZNNi2	BZNNi3
Direct bandgap, ${E}_{opt}^{dir}$ (eV)	4	3.87	3.76	3.85
Indirect bandgap, ${E}_{opt}^{ind}$ (eV)	3.00	3.01	3.03	3.12
Urbach energy, ${E}_{U}$ (eV)	0.56	0.46	0.41	0.35
Steepness parameter,$S$	0.0462	0.0563	0.0631	0.0739
Refractive index,$n$	2.170	2.195	2.218	2.199
Molar refraction, ${R}_{m}$ (cm^3^/mol)	24.6965	24.9463	24.7517	24.9803
Molecular electronic polarizability, ${\alpha }_{em}$ (Å^3^)	9.8002	9.8993	9.8221	9.9128
Dielectric constant,$\varepsilon$	4.7082	4.8199	4.9190	4.8376
Optical dielectric constant,${\varepsilon }_{opt}$	3.7082	3.8199	3.9190	3.8376
Linear dielectric susceptibility,${\chi }_{e}$	0.2951	0.3039	0.3118	0.3053
Reflection loss,${R}_{L}$	0.1362	0.1400	0.1432	0.1405
Transmission coefficient, T	0.7603	0.7545	0.7494	0.7535
Metallization criterion,$M$	0.4472	0.4399	0.4336	0.4387
Electronic polarizability by $n$, ${\alpha }_{{O}^{2-}}\left(n\right)$ (Å^3^)	4.0436	4.0882	4.0593	4.1004
Electronic polarizability by ${E}_{opt}$, ${\alpha }_{{O}^{2-}}\left({E}_{opt}\right)$ (Å^3^)	4.0436	4.0882	4.0593	4.1004
Optical basicity, Λ	1.2570	1.2615	1.2586	1.2627
Numerical aperture, NA	0.3069	0.3105	0.3137	0.3111

**Fig. 12:**
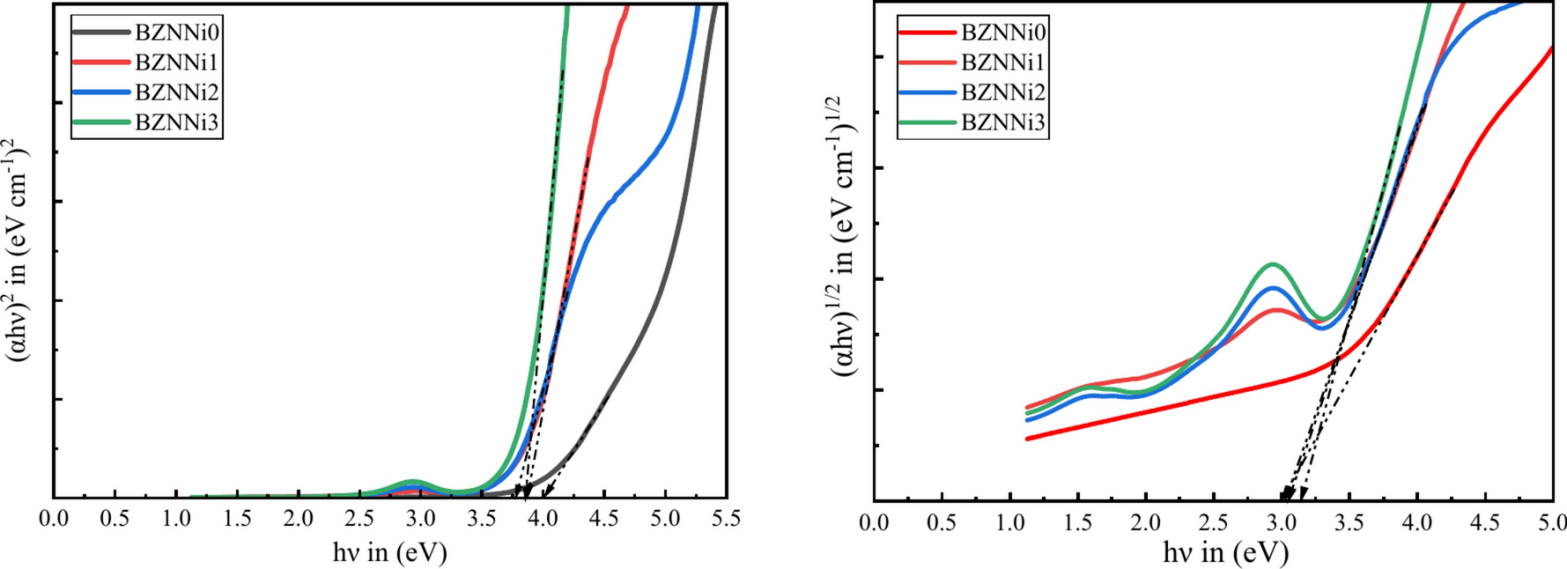
Direct and indirect bandgap of the BZNNi glass series.

The band gap energy (${E}_{g}$) is calculated using Tauc and Menth’s relation and the electron transition using the absorption band^53^, as shown in Eq. 10.

10 $${(\alpha h\nu )}^{n}=C(h\nu -{E}_{opt})$$

where $h\nu$ is the source photon’s energy, ${E}_{opt}$ is the optical band gap, $C$ is constant, and *n* is the transition type index, with *n* = 2 for direct bandgap transitions and *n* = 1/2 for indirect bandgap. Figure 12 depicts the fluctuation in ${(\alpha h\nu )}^{2}$ and ${(\alpha h\nu )}^{1/2}$ with $h\nu$ for prepared glass materials with *n* = 2 and ½^54^. To determine the optical bandgap energies, extrapolate the linear component of the curve to the x-axis at ${(\alpha h\nu )}^{2}$ to 0 and ${(\alpha h\nu )}^{1/2}$ to 0 and displayed in Table 5. Resulting the direct bandgap (${E}_{opt}^{dir}$) of samples decreased from 4.00 to 3.76 eV as the concentration of the NiO increased till BZNNi2 due to increase in the density of the glass network and increased to 3.85 eV for BZNNi3 due to decrease in the density in the glass. But the indirect bandgap (${E}_{opt}^{ind}$) varies between 3.00 and 3.12 eV^51^.

### Urbach energy (${{\varvec{E}}}_{{\varvec{U}}}$) and steepness parameter (S)

**Fig. 13:**
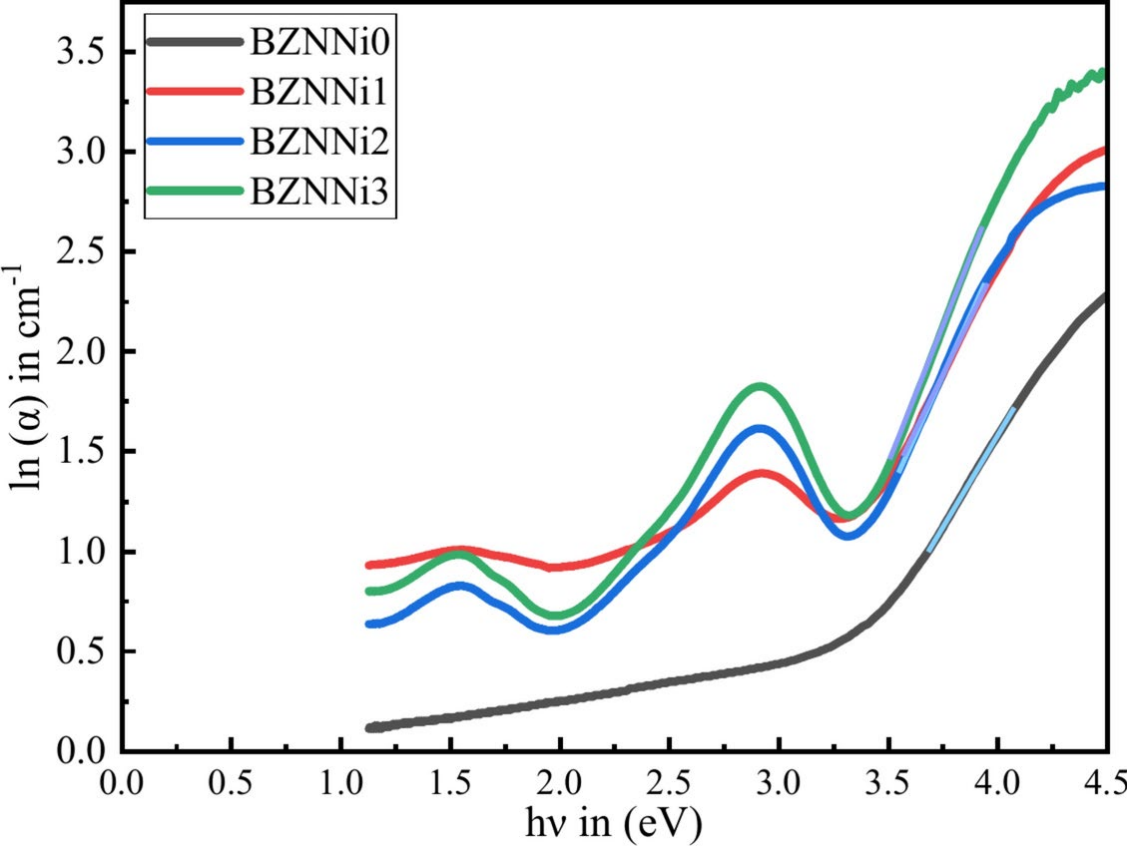
Urbach energy of the BZNNi glass series.

UV spectroscopy reveals a few cases of localized band gap defects called Urbach energy. The excited electrons are blocked by these defect states, which keeps them from direct transition into the conduction band. Defect states present in glass are the reason for the tail that is commonly observed in absorption spectra, and it extends into the forbidden gap. This tail’s width, which is associated with localized band gap defects is measured by the Urbach energy (${E}_{U}$)^40^. The ${E}_{U}$ value can be determined by using the below Eq. 11.

11 $$\alpha =C{e}^{h\nu /{E}_{U}}$$

where $C$ is the constant, $\nu$ is the radiation frequency, $h$ is the Plank’s constant, ${E}_{U}$ is the Urbach energy. Figure 13 shows a relationship between ln(α) and $h\nu$. Urbach energy is the reciprocal of the slope that is obtained by the linear part of the plot. As the concentration of the NiO increased, the Urbach energy decreased from 0.56 to 0.35, leading to the glass’s compactness resulting in fewer defects which verifies with N4 parameter. The obtained values of ${E}_{U}$ are noted in Table 5.

The steepness parameter $S$ is the widening of the optical absorption edge caused by electron–phonon or exciton-phonon interaction^55^. The formula shown in Eq. 12 can be used to compute the steepness parameter.

12 $$S=\frac{{k}_{B}T}{{E}_{U}}$$

where $T$ is the room temperature (in K) and ${k}_{B}$ is the Boltzmann constant. The decrease in the value of the band gap defects found in the glass system determines the increase in $S$ from 0.046 to 0.073. The calculated values are displayed in Table 5.

### Refractive index (${\varvec{n}}$), molar refraction (${{\varvec{R}}}_{{\varvec{m}}}$) and molecular electronic polarizability (${\boldsymbol{\alpha }}_{{\varvec{e}}{\varvec{m}}}$)

The interaction of incident light with the atomic electrons of the anion and cation of oxides in the glass can influence the refractive index of oxide glass. Dimitrov and Sakka^56^ presented a method for determining the refractive index of glasses using the optical band gap, which is given by Eq. 13.

13 $$\frac{({n}^{2}-1)}{({n}^{2}+2)}=\left(1-{\left(\frac{{E}_{Opt}}{20}\right)}^\frac{1}{2}\right)$$

**Fig. 14:**
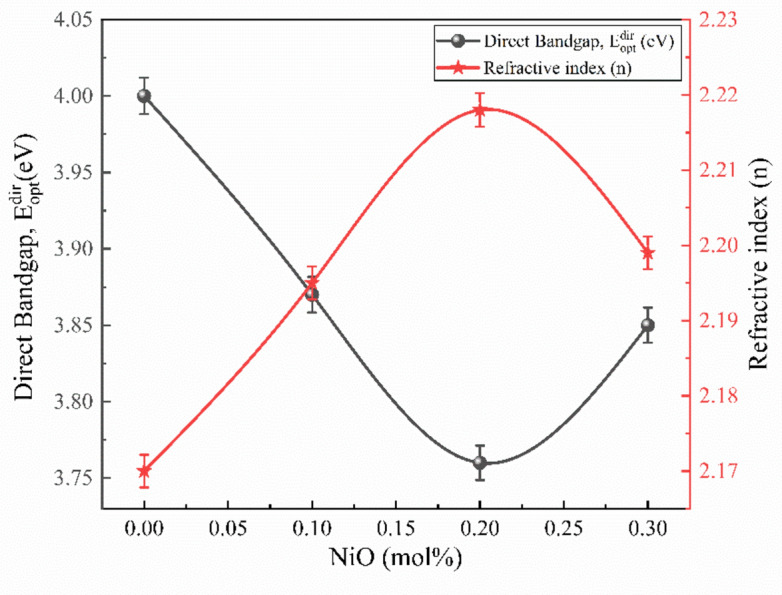
Comparison with ${E}_{opt}^{dir}$ and refractive index ($n$) of the BZNNi glass series.

Table 5 displays the calculated values of refractive index of the glass. As the Ni^2+^ concentration increases, the refractive index of the BZNNi series glass rises 2.17 to 2.218 until 0.2 mol% but decreases to 2.199 in 0.3 mol%, opposite to the energy band gap of the glasses illustrated in Fig. 14. This variation of the potential for localized excitations of the NiO and the electron oxide polarizability of glass will influence changing the refractive index of the glass.

The molar refraction (${R}_{m}$) of isotropic materials is determined using the Lorentz-Lorenz equation, which corresponds to molar volume (${V}_{m}$) and refractive index ($n$), as seen in Eq. 14. The calculated values are shown in Table 5.

14 $${R}_{m}=\left(\frac{{n}^{2}-1}{{n}^{2}+2}\right)({V}_{m})$$

Equation 15 illustrates the relationship between molecular electronic polarizability and molar refraction.

15 $${2.52 \times \alpha }_{em}={R}_{m}$$

The molar refractivity index (${R}_{m}$) and the molar electronic polarizability (${\alpha }_{em}$) have a linear correlation. The values vary from 24.6965 to 24.9803 cm^3^mol^-1^ and from 9.8002 to 9.9128 Å^3^ respectively^25^.

### Dielectric constant (${\varvec{\varepsilon}}$), optical dielectric constant (${{\varvec{\varepsilon}}}_{{\varvec{o}}{\varvec{p}}{\varvec{t}}}$) and linear dielectric susceptibility (${{\varvec{\chi}}}_{{\varvec{e}}}$)

The refractive index and the dipole orientation contribution to polarizability for any glass system have a strong relationship with the dielectric constant ($\varepsilon$), optical dielectric constant (${\varepsilon }_{opt}$), and linear dielectric susceptibility (${\chi }_{e}$) values and the relations are as follows:

16 $$\varepsilon ={n}^{2}$$

17 $${\varepsilon }_{opt}={n}^{2}-1$$

18 $${\chi }_{e}=\frac{\varepsilon -1}{4\pi }$$

**Fig. 15:**
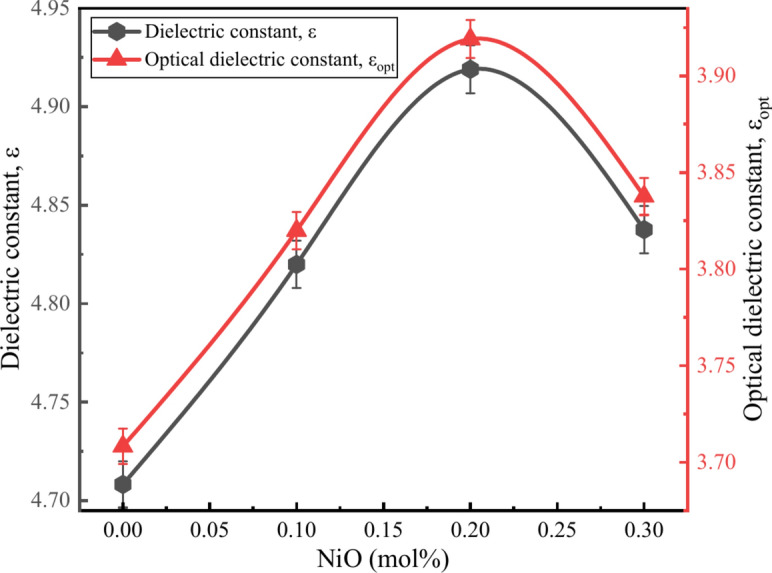
Relationship between $\varepsilon$ and ${\varepsilon }_{opt}$ of the BZNNi glass series.

Table 5 and Fig. 15 shows the values of $\varepsilon$ and ${\varepsilon }_{opt}$ varied from 4.7082 to 4.9190 and from 3.7082 to 3.9190 respectively. The values of ${\chi }_{e}$ varied from 0.2951 to 0.3118 which has a linear relationship with $n$ values. This relationship confirmed the theoretical assumption that the ${\chi }_{e}$ values linearly depended on the $n$ values^30,57^.

### Reflection loss (${{\varvec{R}}}_{{\varvec{L}}}$), transmission coefficient (${\varvec{T}}$) and metallization criteria (${\varvec{M}}$)

The following relationship from Eqs. 19, 20, and 21 is used to get the reflection loss (${R}_{L}$), transmission coefficient ($T$), and metallization criterion ($M$)^25,57^.

19 $${R}_{L}={\left(\frac{n-1}{n+1}\right)}^{2}$$

20 $$T=\frac{2n}{{n}^{2}+1}$$

21 $$M=1-\frac{{R}_{m}}{{V}_{m}}$$

**Fig. 16:**
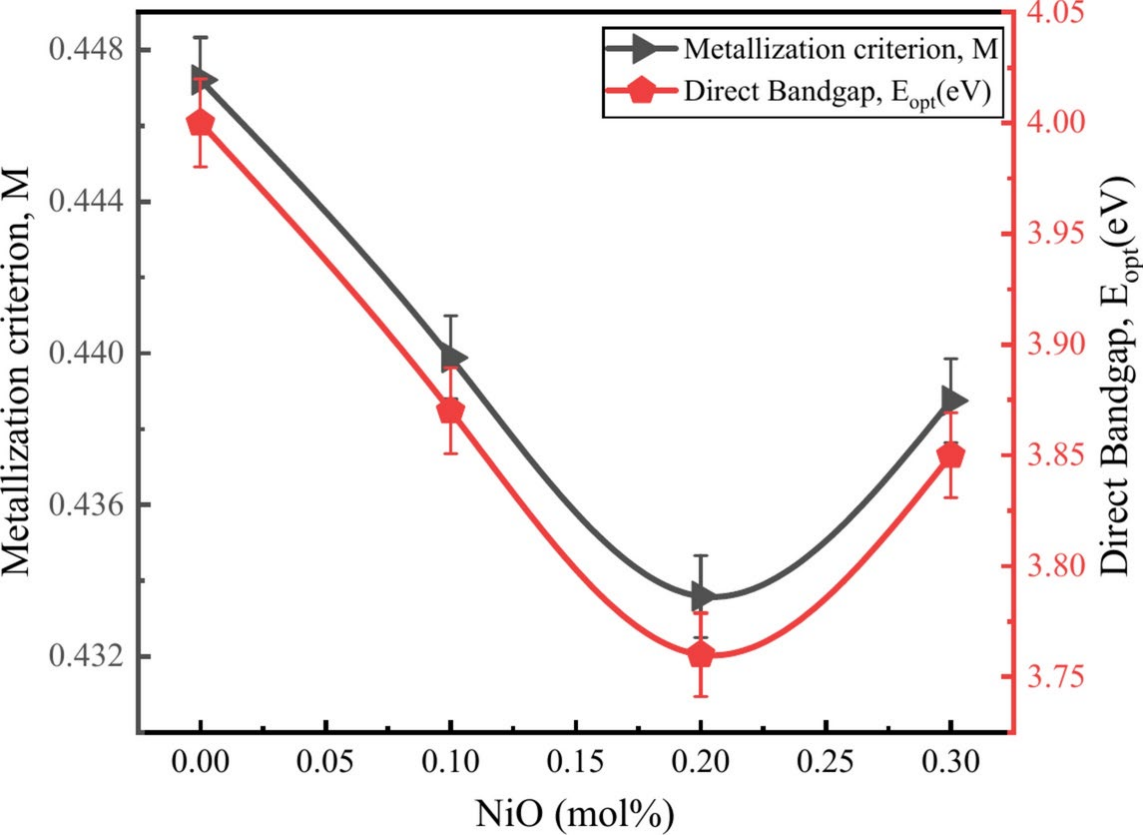
Relationship between $M$ and ${E}_{opt}^{dir}$ of the BZNNi glass series.

Table 5 shows the transmission coefficient ($T$) and reflection loss (${R}_{L}$) from the surface of the glass^53,57^. The values shown in Table 5 have an inverse relationship, supporting the theory that transmission is inversely proportional to reflection. The metallization criterion (M) varies from 0.4472 to 0.4387 for BZNNi glass. Further, insulators are materials with a high M value which is close to 1, whereas more metallic materials have a value of M close to 0^25,33^. The change in the direct bandgap is attributed to the change in metallization shown in Fig. 16. It appears that in the current glasses, BZNNi2 glass has a metallic nature.

### Electronic oxide ion polarizability

**Fig. 17:**
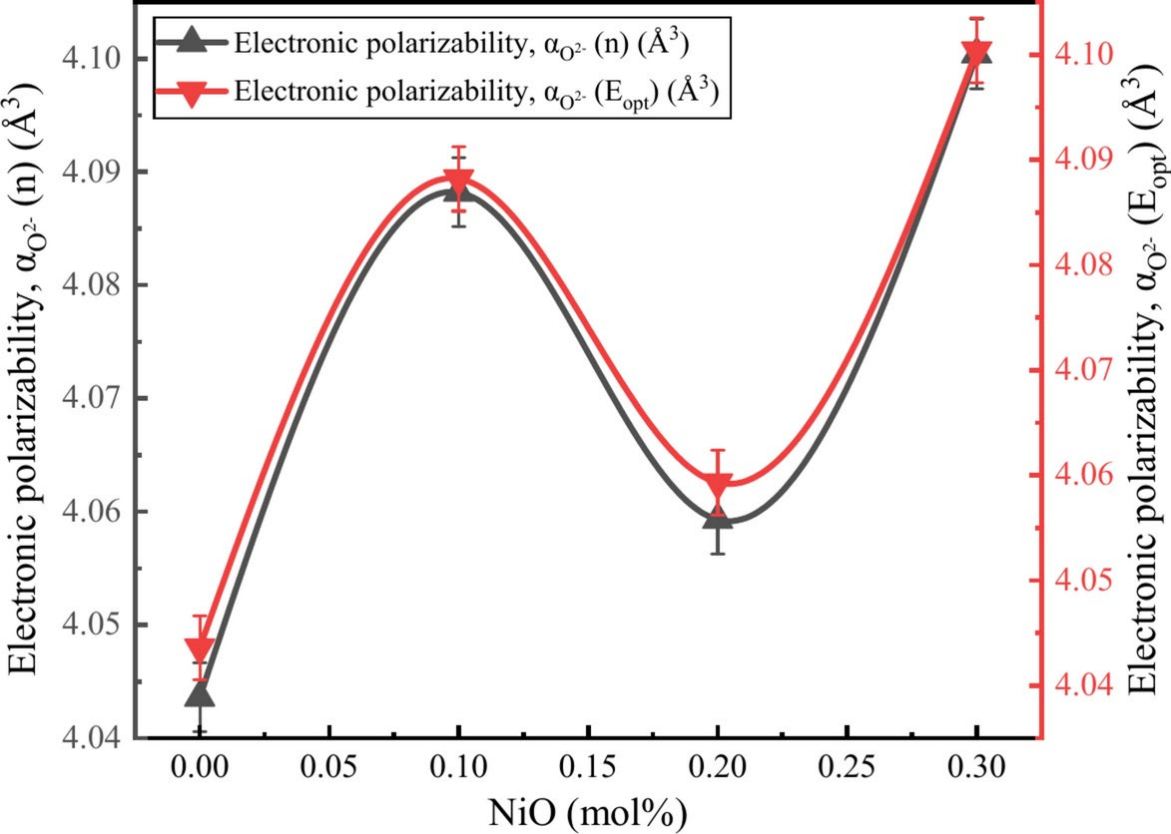
Relationship between ${\alpha }_{{O}^{2-}}\left(n\right)$ and ${\alpha }_{{O}^{2-}}\left({E}_{opt}\right)$ of BZNNi glass series.

The electronic polarizability of oxide ions can be determined using the refractive index ($n$) and optical band gap (${E}_{opt}$).

22 $${\alpha }_{{O}^{2-}}\left(n\right)=\left[\left(\frac{{V}_{m}}{2.52}\right)\left(\frac{{n}^{2}-1}{{n}^{2}+2}\right)-\sum {\alpha }_{cat}\right]/[{N}_{{O}^{2-}}]$$

23 $${\alpha }_{{O}^{2-}}\left({E}_{opt}\right)=\left[\left(\frac{{V}_{m}}{2.52}\right)\left(1-\sqrt{\frac{{E}_{opt}}{20}}\right)-\sum {\alpha }_{cat}\right]/[{N}_{{O}^{2-}}]$$

where $\sum {\alpha }_{cat}$ stands for molar cation polarizability and ${N}_{{O}^{2-}}$ stands for the number of oxide ions in the stoichiometry ratio of the glass a*B*_*2*_*O*_*3*_ – b*ZnO* – c*Na*_*2*_*O* – d*NiO*, where $\sum {\alpha }_{cat}$ is given by a × 2×${\alpha }_{{B}^{3+}}$  + b × 1×${\alpha }_{{Zn}^{2+}}$  + c × 1×${\alpha }_{{Na}^{+}}$  + d × 1 × ${\alpha }_{{Ni}^{2+}}$, the values of ${\alpha }_{{B}^{3+}}$= 0.002 Å^3^, ${\alpha }_{{Zn}^{2+}}$= 0.283 Å^3^, ${\alpha }_{{Na}^{+}}$= 0.181 Å^3^, and ${\alpha }_{{Ni}^{2+}}$= 0.266 Å^3^ whereas the ${N}_{{O}^{2-}}$ is given by a × 3 + b × 1 + c × 1 + d × 1^58,59^. Table 5 shows a varying pattern in the polarizability of oxide ions to that of molecular electronic polarizability. The calculated oxide ion polarizability is based on the refractive index ${\alpha }_{{O}^{2-}}\left(n\right)$ and optical band gap ${\alpha }_{{O}^{2-}}\left({E}_{opt}\right)$. Both exhibit similar behavior, ranging from 4.0436 Å^3^ to 4.1004  Å^3^. Figure 17 displays the calculated values of the refractive index’s electronic oxide polarizability and optical band gap with NiO concentration.

### Optical basicity and numerical aperture of glass.

Optical basicity is defined as the ability to transfer an ion’s negative charge. Equation 24 can be used to determine it using electronic oxide polarizability.

24 $$\Lambda =1.67\left(1-\frac{1}{{\alpha }_{{O}^{2-}}}\right)$$

**Fig. 18:**
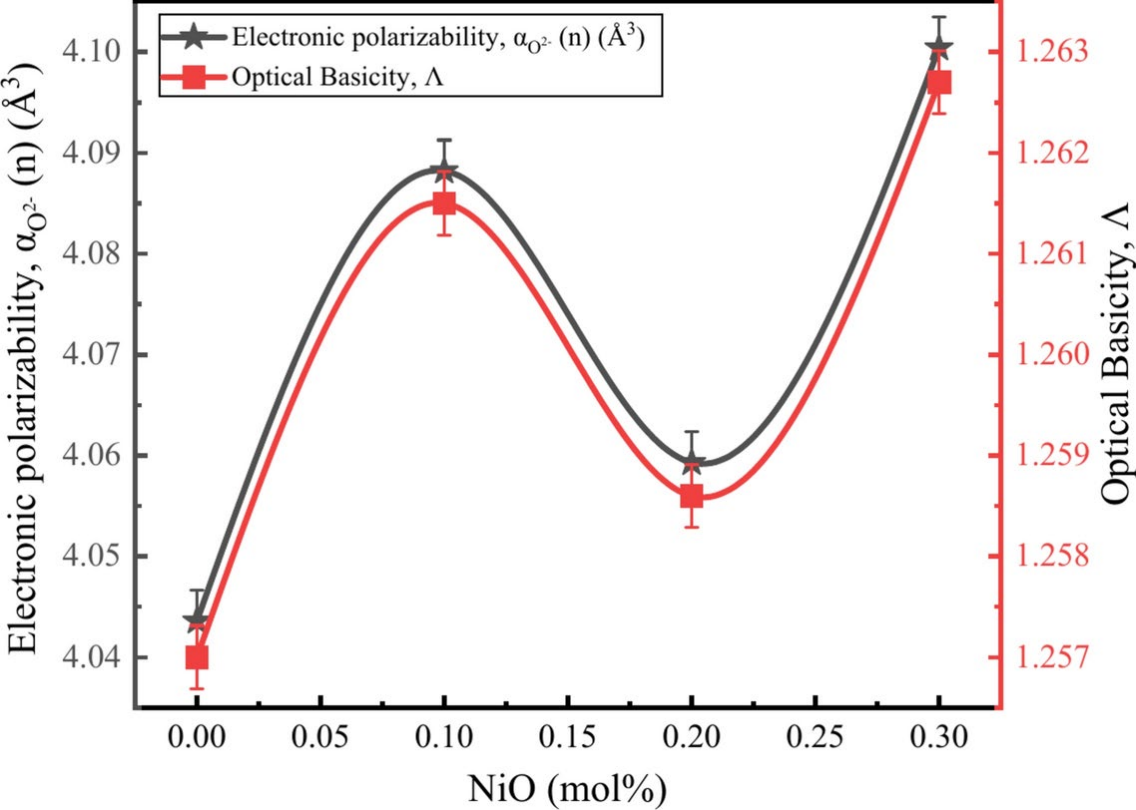
Relationhip between $\boldsymbol{\Lambda }$ and ${\boldsymbol{\alpha }}_{{{\varvec{O}}}^{2-}}\left({\varvec{n}}\right)$ of BZNNi glass series.

The optical basicity of the BZNNi glasses is varied from 1.2570 to 1.2627 as shown in Table 5. The optical basicity $\Lambda$ of the of the glass is varied with respect to the ${\alpha }_{{O}^{2-}}\left(n\right)$ as the concentration of the NiO increases in the BXNNi glasses, and it is directly related to the electron oxide polarizability as shown in Fig. 18.

The numerical aperture (NA) of the prepared glass is calculated using the following Eq. 25.

25 $$NA={n[2\Delta ]}^{1/2}$$

where $\Delta$ is the fractional RI change (0.01)^25^ and $n$ is the refractive index of the glass. If the NA values range between 0.13 and 0.5, these glasses can be used as the core material in optical fiber cable. These prepared BZNNi glasses are good alternates for optical fiber cable as core materials because of their enhanced numerical aperture which ranges from 0.3069 to 0.3137 and refractive index which is directly proportional to each other. The calculated values are displayed in Table 5.

### Photoluminescence spectra

The present glass matrix with NiO is expected to contain octahedral and tetrahedral coordination sites for Ni^2+^ ions (3d^8^)^13^. The luminescence of sodium zinc borate glasses doped with Ni^2+^ ions is possibly related to the d–d optical transitions. In octahedral symmetry, the energy levels of Ni^2+^ ions were found to be ^3^A_2g_(F) → ^3^T_2g_(F), ^3^A_2g_(F) → ^3^T_1g_(F), and ^3^A_2g_(F) → ^3^T_1g_(P). In addition to the three spin-allowed transitions, a spin-forbidden transition ^3^A_2g_(F) → ^1^E_g_(D) can also be seen through the luminescence spectra^60–62^.

**Fig. 19:**
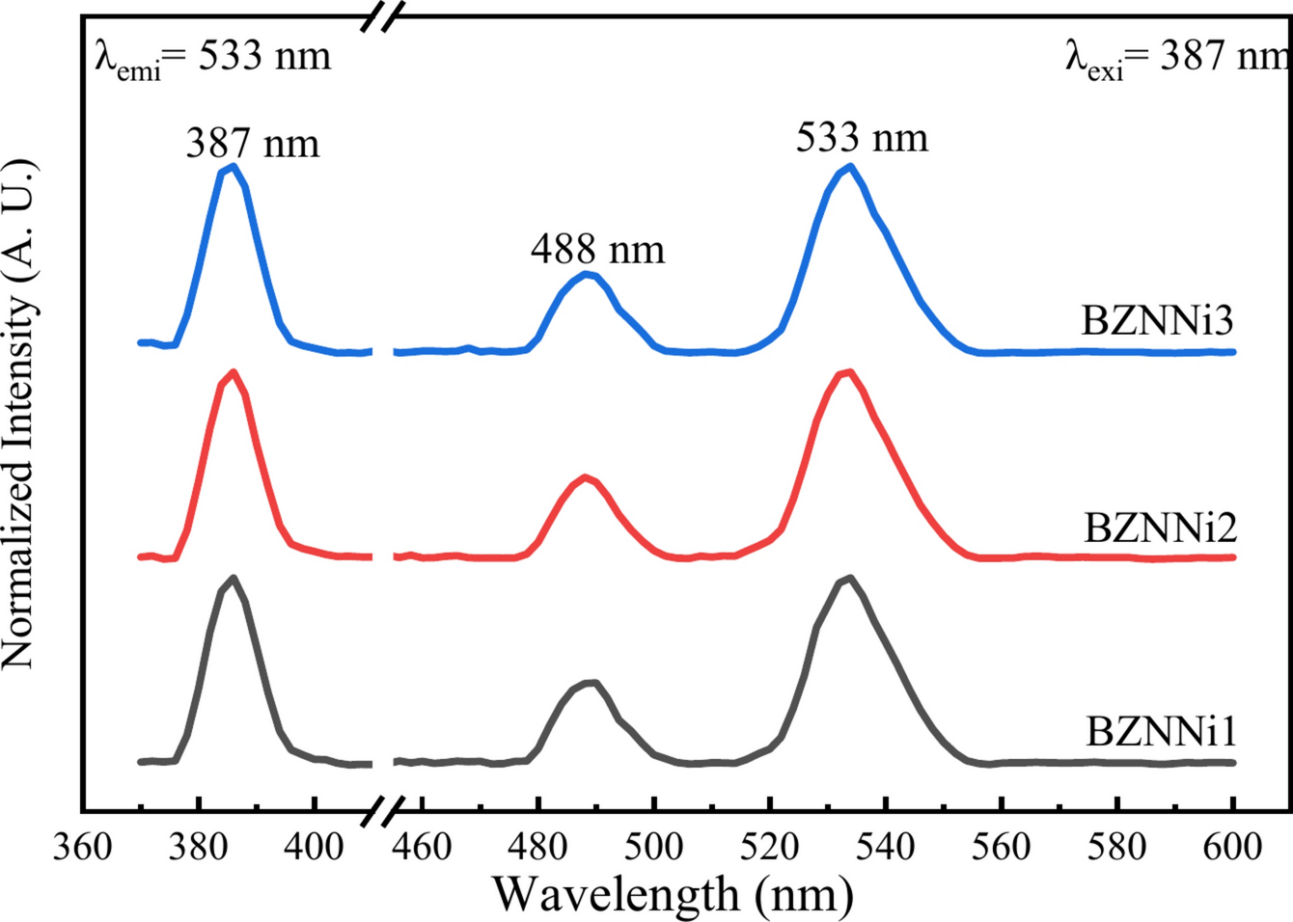
Photoluminescence spectra of the BZNNi glass series.

**Fig. 20:**
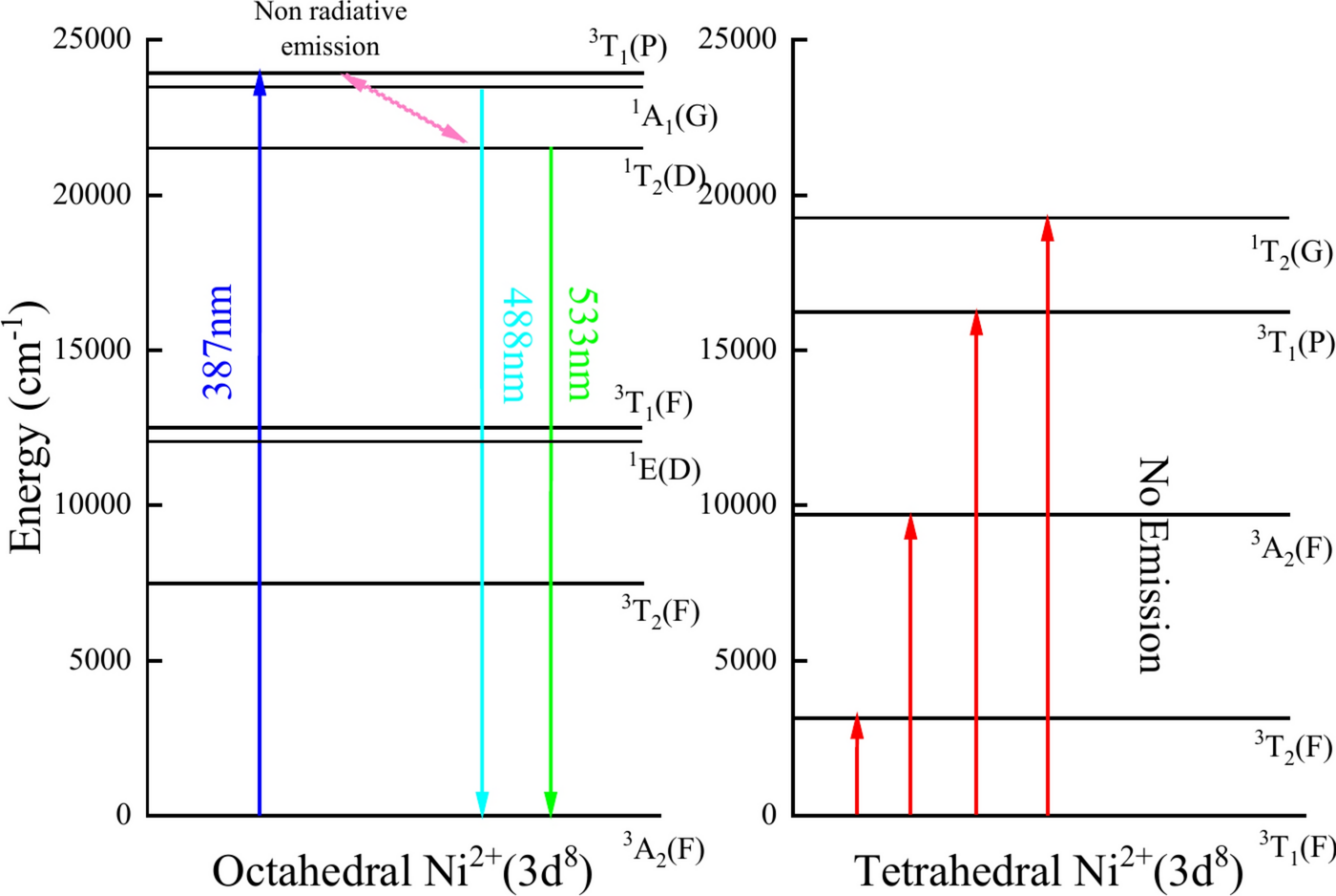
Energy level diagram of the Ni^2+^(3d^8^).

The photoluminescence spectra of BZNNi glasses, which were recorded at room temperature with an excitation wavelength of 387 nm (^3^A_2g_(F) → ^3^T_1g_(P)), are shown in Fig. 19. They exhibit two strong broad emission bands that are centered at around 488 nm and 533 nm. The cyan emission peak at 488 nm was attributed to an energy level transition of ^1^A_1_(G) → ^3^A_2g_(F)^63^. The green emission peak around 533 nm is associated with the ^1^T_2g_(D) → ^3^A_2g_(F) transition of Ni^2+^ as shown in Fig. 19^63,64^. Figure 20 shows the energy level diagram of Ni^2+^ doped sodium zinc borate glasses^61,63,64^.

**Fig. 21:**
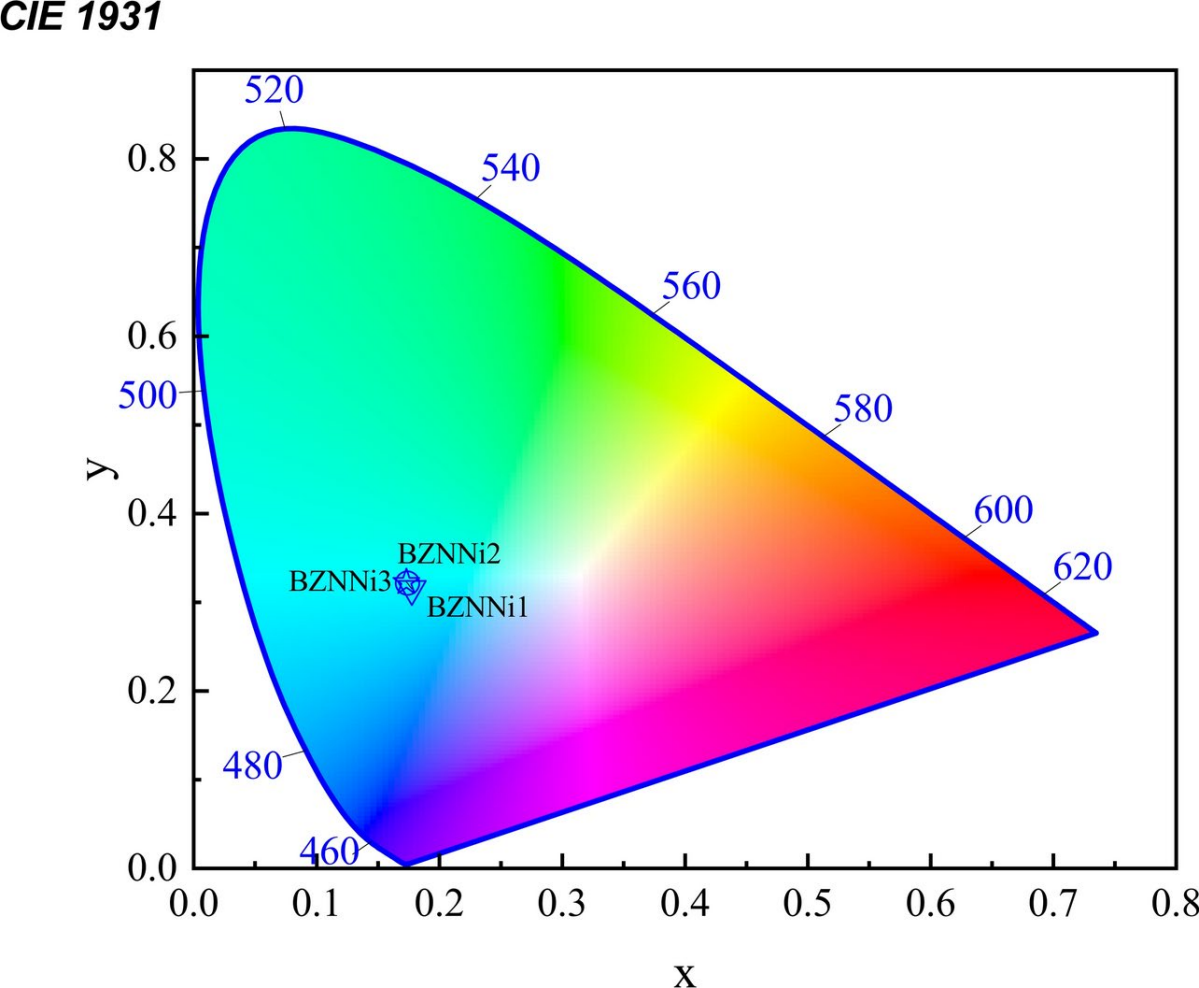
CIE Chromacity analysis of the BZNNi glass series.

A CIE coordinate calculator is used to determine the chromaticity coordinates (x_n_, y_n_) of BZNNi glass materials. Higher orders of covalence, including NiO, are found in the internal structure of the current BZNNi glass materials. It appears to be possible to emit green light based on the observed intensity magnitudes within the visible emission region. In Fig. 21, the color coordinate region is represented by the display of the symbol within the CIE chromaticity diagram. The calculated coordinates around x_1_ (0.177), y_1_ (0.315), x_2_ (0.174), y_2_ (0.321), x_3_ (0.173), and y_3_ (0.323) for BZNNi1, BZNNi2, BZNNi3 glasses respectively, that correlate to the CIE figure indicate that green emission in these glasses is adequate for optoelectronic applications^40,65^.

## Conclusion

The present study shows that incorporating NiO into the Na_2_O-ZnO-B_2_O_3_glass matrix significantly changes its structural and optical properties. XRD study confirmed the non-crystalline structure of the prepared glasses. Morphological and elemental analysis is studied using SEM images and EDS spectra. Further, FTIR and Raman spectra including its deconvolution demonstrated by the N4 parameters from 0.54 to 0.63 explain the conversion of BO_3_ units to BO_4_ units due to the addition of NiO in the glass system. The absorption bands of NiO were seen in 425 nm and 800 nm corresponding to ^3^A_2g_ (F) → ^3^T_1g_ (P)) and (^3^A_2g_ (F) → ^1^E_g_(^3^F)) transitions respectively in the UV–Vis spectrum. The optical characteristics revealed the variation in the direct bandgap from 4.00 to 3.76 eV with increasing NiO content. Furthermore, the drop in Urbach energy from 0.56 to 0.35 indicated that increasing NiO concentration showed improved network compactness and structural rigidity verified with the N4 parameter. The optical parameters were calculated to correspond to the optical bandgap value. Electron ion polarizability and metallization criteria values ranging from 4.0436 to 4.1004 and from 0.4472 to 0.4336 respectively showed the ionic and metallic nature of the prepared glasses. The PL spectra exhibit cyan (488 nm) emission with ^1^A_1_(G) → ^3^A_2g_(F) transition and green (533 nm) emission with ^1^T_2g_(D) → ^3^A_2g_(F) transition attributed to d-d transitions of Ni^2+^. The observed green emission in CIE Chromacity coordinates results and calculated optical properties suggest that the prepared glasses is useful for various applications in optoelectronic devices.

## Data Availability

The datasets used and/or analysed during the current study available from the corresponding author on reasonable request.
